# Mineralogy and mineral chemistry of the ABM replacement-style volcanogenic massive sulfide deposit, Finlayson Lake district, Yukon, Canada

**DOI:** 10.1007/s00126-023-01217-4

**Published:** 2023-10-06

**Authors:** Nikola Denisová, Stephen J. Piercey, Markus Wälle

**Affiliations:** 1 Department of Earth Sciences, Memorial University of Newfoundland, St. John’s, NL A1B 3X5 Canada; 2 CREAIT, CRC and CFI Services (CCCS), Bruneau Centre for Research and Innovation, Memorial University of Newfoundland, St. John’s, NL A1C 5S7 Canada

**Keywords:** ABM deposit, Finlayson Lake district, Replacement-style VMS, In situ sulfide mineral chemistry

## Abstract

The ABM deposit is a bimodal-felsic, replacement-style volcanogenic massive sulfide deposit (VMS) that is hosted by back-arc affinity rocks of the Yukon–Tanana terrane in the Finlayson Lake VMS district, Yukon, Canada. Massive sulfide zones occur as stacked and stratabound lenses subparallel to the volcanic stratigraphy, surrounded by pervasive white mica and/or chlorite alteration. Remnant clasts of volcanic rocks and preserved bedding occur locally within the massive sulfide lenses and indicate that mineralization formed through subseafloor replacement of pre-existing strata. Three mineral assemblages occur at the ABM deposit: (1) a pyrite–chalcopyrite–magnetite–pyrrhotite assemblage that is associated with Cu–Bi–Se–Co-enrichment and occurs at the center of the massive sulfide lenses; (2) a pyrite–sphalerite assemblage, which occurs more commonly towards lens margins and is enriched in Zn–Pb–Ag–Au–Hg–As–Sb–Ba; and (3) a minor assemblage comprising chalcopyrite–pyrrhotite–pyrite stringers associated with pervasive chlorite alteration, which occurs mostly at the sulfide lens margins. Petrographic observations of preserved primary, zone refining, and metamorphic textures in combination with in situ geochemistry show that the pyrite–sphalerite assemblage formed at lower temperatures (< 270 °C) than the other two mineral assemblages (~ 270–350 °C), and that mineral chemistry in all mineral assemblages was affected by greenschist facies metamorphism, although the effects are limited to recrystallization, small-scale remobilization (< 1 m) and trace element redistribution.

**Supplementary Information:**

The online version contains supplementary material available at 10.1007/s00126-023-01217-4.

## Introduction

Volcanogenic massive sulfide (VMS) deposits are critical sources of base and precious metals (e.g., Galley et al. 2007); however, what controls the grade and tonnage of the mineralization is not completely understood. In a subset of VMS deposits, subseafloor replacement is an important process, where a greater proportion of the mineralizing fluids is precipitated in the subsurface (Doyle and Allen 2003; Piercey 2015). These types of deposits are interpreted to have had higher precipitation efficiency than exhalative-style deposits, resulting in deposits with higher tonnages and/or higher grades relative to those precipitated on the seafloor (Doyle and Allen 2003; Piercey 2015). Further, zone refining, the dissolution of existing mineralization by higher-temperature fluids and precipitation of new high-temperature mineralization, is one of the critical mechanisms responsible for increasing the grades of massive sulfide mineralization in both exhalative- and replacement-style deposits (Eldridge et al. 1983; Ohmoto 1996). In many ancient deposits, however, distinguishing textural features of emplacement origin and zone refining are commonly obscured due to post-VMS overprinting, metamorphism, and deformation (Lafrance et al. 2020). This creates a unique challenge in VMS deposit research: recognition of primary exhalative- and replacement-related textures versus those imposed by subsequent post-VMS formation events (e.g., Craig and Vokes 1992; Larocque and Hodgson 1995; Lafrance et al. 2020).

Past studies have been successful in identifying replacement-style VMS mineralization in the ancient record, including documenting the mineralizing fluid evolution, metal sources, and the impact of metamorphic and structural overprinting (Larocque and Hodgson 1995; Genna et al. 2014; Brueckner et al. 2016; Vikentyev et al. 2017). However, the effects of metamorphism on VMS mineralization, even at low metamorphic grades, have not been yet fully resolved. Primary geochemical signatures of some elements in sulfides can be influenced by metamorphic overprinting (Lockington et al. 2014; Genna and Gaboury 2015; George et al. 2016). Further, correctly interpreting primary versus secondary textures from field to microscopic scale is critical for understanding the relative roles of VMS-related exhalation, replacement, and zone refining during ancient VMS formation, versus secondary metamorphic/structural influences on mineralization, mineral textures, and assemblages (e.g., Layton-Matthews et al. 2008; Brueckner et al. 2014, 2016; Carvalho et al. 2018; Martin et al. 2018; Cugerone et al. 2021). Deciphering these effects is important for understanding the distribution of economic (e.g., Ag and Au), critical (e.g., Co, Se, or Sn), and potentially deleterious (e.g., As and Cd) metals.

The bimodal-felsic ABM deposit is a replacement-style VMS deposit with a total (geological) resource of 19.1 Mt at 6.3 wt. % Zn, 0.9 wt. % Cu, 1.9 wt. % Pb, 1.4 g/t Au and 148 g/t Ag (van Olden et al. 2020). The deposit is located in the Finlayson Lake VMS district, Yukon, Canada, which contains > 40 Mt of polymetallic VMS mineralization with varying styles of deposits hosted by arc and back-arc rocks of the Yukon–Tanana and Slide Mountain terranes (Peter et al. 2007). Following a drilling program in 2015, the massive sulfide mineralization at the ABM deposit was re-interpreted as replacement style based on lithofacies, textural, and structural studies (van Olden et al. 2020; Denisová and Piercey 2022; Manor et al. 2022a). Previous work on the massive sulfide mineralization at the ABM deposit focused on the distribution and sources of Se (Layton-Matthews et al. 2008, 2013), but comprehensive research has not been presented on the mineralization facies, mineralogy, textures, metal residence, and genesis of the massive sulfide mineralization in the deposit.

This contribution is third in a series of studies that focus in detail on the ABM deposit; the first study described in detail the lithostratigraphic setting and tectonomagmatic environment hosting the deposit; the second study focused on the hydrothermal alteration and its evolution. This study presents new, previously unpublished results of textural and mineralogical studies derived from drill core observations, assay data, 3D modeling, petrography and paragenetic studies, electron probe microanalysis (EPMA), and laser ablation inductively coupled plasma mass spectrometry (LA-ICP-MS). We discuss the timing and evolution of the mineralization and the characteristics and potential sources of mineralizing fluids in the ABM deposit. Further, we distinguish primary subseafloor VMS-related mineralogical and geochemical signatures from those related to greenschist-facies metamorphic overprinting. The results herein contribute to our understanding of formation of massive sulfides in ancient, metamorphosed replacement-style VMS deposits.

### Regional geology

**Fig. 1:**
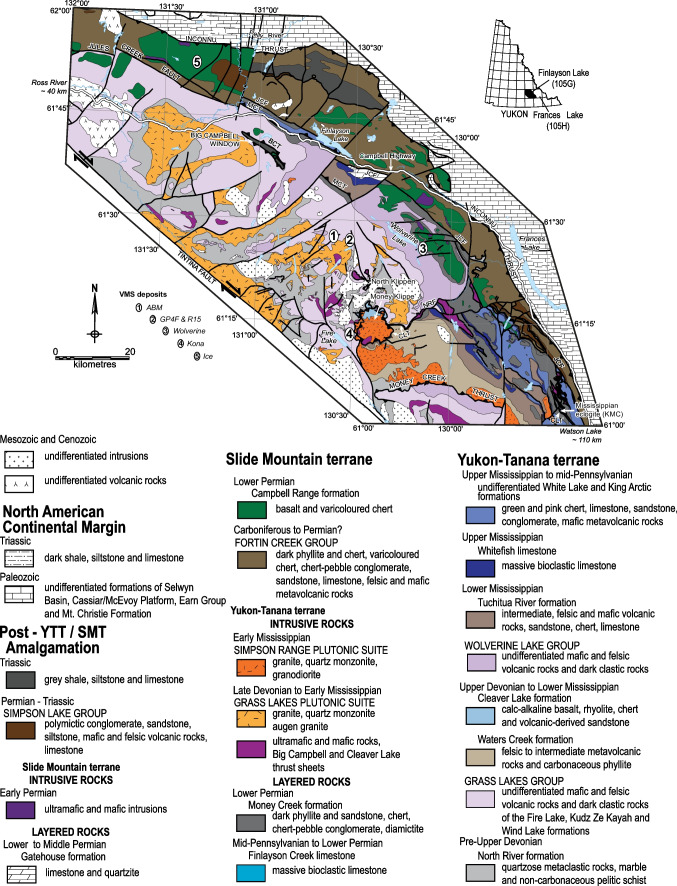
Regional setting of the Finlayson Lake district (modified after Murphy et al. 2006; Piercey et al. 2016; Manor and Piercey 2018). Numbers mark the positions of known VMS deposits in the region. BCT = Big Campbell thrust; CLT = Cleaver Lake thrust; JCF = Jules Creek fault; MCT = Money Creek thrust; NRF = North River thrust

The Finlayson Lake VMS district is a dismembered block of the Yukon–Tanana and Slide Mountain terranes that developed along the western margin of Laurentia from the Devonian to the Permo-Triassic (Fig. 1; Colpron et al. 2006; Nelson et al. 2006; Piercey et al. 2006). The Yukon–Tanana terrane in the district comprises a poly-deformed and metamorphosed pre-Late Devonian continental margin assemblage (Piercey and Colpron 2009) that is overlain by three unconformity-bound Late Devonian to Middle to Late Permian continental arc, back-arc, and ocean basin–related volcanic-sedimentary sequences (Mortensen and Jilson 1985; Mortensen 1992; Colpron et al. 2006; Murphy et al. 2006). Metamorphism and deformation in the district are interpreted to be a result of a Middle Jurassic–Early Cretaceous mid-crustal tectonometamorphic event, which comprised ductile deformation and moderate temperature-high pressure metamorphism (Staples et al. 2014). The core of the Finlayson Lake district reached amphibolite facies metamorphic grade, which transitions to lower greenschist facies further from the center of the district (Murphy et al. 2006). The Big Campbell thrust sheet is by volume the largest and structurally deepest block in the Finlayson Lake district (Fig. 1) and hosts four VMS deposits (Fig. 1; Murphy et al. 2006; Peter et al. 2007). The Grass Lake group is composed of three units (Fig. 1). The Fire Lake formation hosts the Kona Cu–Co–Au mafic–siliciclastic VMS deposit (Piercey et al. 2001a; Sebert et al. 2004; Murphy et al. 2006; Peter et al. 2007). The Kudz Ze Kayah formation is interpreted to be coeval to the Fire Lake formation (Manor et al. 2022b); it comprises dominantly felsic volcanic and sedimentary rocks with back-arc geochemical affinities (Piercey et al. 2001b; Murphy et al. 2006; Denisová and Piercey 2022; Manor et al. 2022a). The Wind Lake formation sits conformably atop the Kudz Ze Kayah formation (Piercey et al. 2002). All rocks in the Grass Lakes group are intruded by the Grass Lakes plutonic suite at ca. 361 Ma (Piercey et al. 2001b, 2003; Manor et al. 2022b).

Three known VMS deposits are hosted within the Kudz Ze Kayah formation: the ABM, GP4F, and R15 deposits. The ABM deposit is located about 25 km south of Finlayson Lake and the Robert Campbell Highway (Fig. 1). The GP4F deposit is situated roughly 5 km SE from the ABM deposit (Fig. 1) and sits ~ 500–600 m stratigraphically below the ABM deposit (Peter et al. 2007; Manor et al. 2022a). The R15 deposit occurs immediately along strike east of the GP4F deposit and occupies the same stratigraphic position (MacRobbie and Holroyd, unpub. data). The mineralization style at the R15 (MacRobbie and Holroyd, unpub. data) deposit is described as similar to the GP4F deposit (Boulton 2002). Subseafloor replacement is interpreted to be the primary mineralization style in all three deposits (Peter et al. 2007; van Olden et al. 2020; Denisová and Piercey 2022; Manor et al. 2022a). The ABM deposit is hosted by rocks formed at ca. 362.82 ± 0.12 Ma (Manor et al. 2022a).

### Local geology

**Fig. 2:**
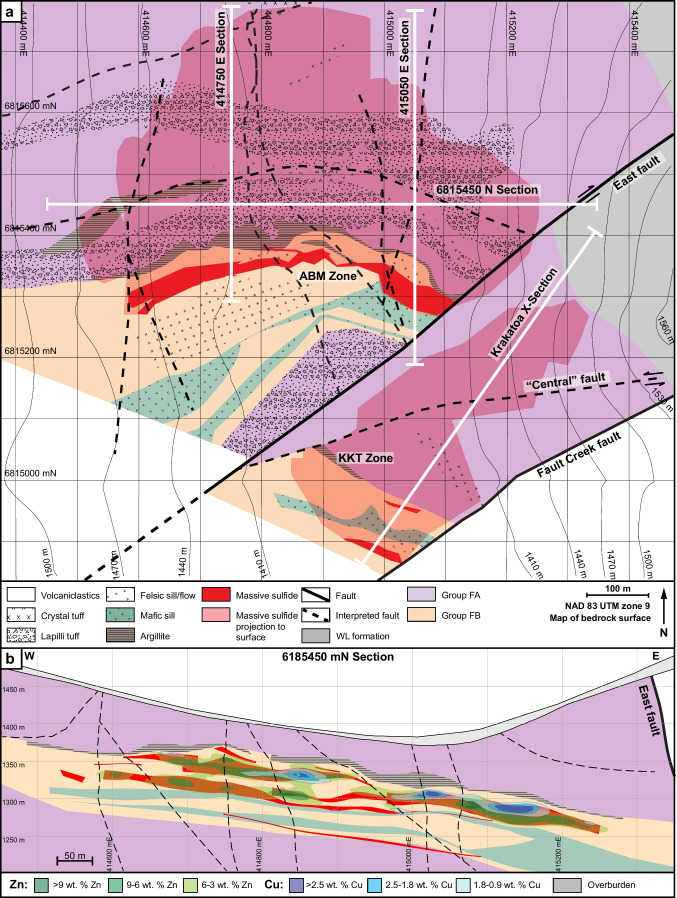
Local geology of the ABM deposit. **a** Geological map with units constructed using drilling data and 3D models. Section lines displayed. Upward projections of maximum known extent of mineralization displayed. Note that lithofacies are displayed using patterns and geochemical groups using colors. Projection grid is NAD 83 UTM zone 9. **b** Section through the ABM zone of the ABM deposit running W–E, looking north with simplified lithofacies and lithogeochemistry displayed. Contours of Zn and Cu content are overlayed on the simplified stratigraphy

The upper Kudz Ze Kayah formation is interpreted to have been deposited in a back-arc environment (Piercey et al. 2001b, 2002) in a lower order basin with an active volcanic center (Denisová and Piercey 2022). The ABM deposit is hosted within a volcanosedimentary package that occupies the upper ~ 350 m of the Kudz Ze Kayah formation. The stratigraphy dips between 20 and 30° to the NNE, and field relationships indicate no fault repetition or major folding (van Olden et al. 2020; Denisová and Piercey 2022; Manor et al. 2022a). The East fault is interpreted to be a re-activated transform fault (Fig. 2a; van Olden et al. 2020) that was originally part of a set of interconnected synvolcanic normal faults that accommodated extension within the basin and acted as magma conduits (Denisová and Piercey 2022).

The upper Kudz Ze Kayah formation is divided into three sequences (Fig. 2b) with different geochemical characteristics based on immobile element systematics (e.g., Zr/Al_2_O_3_, Al_2_O_3_/TiO_2_, Nb/Ta; Denisová and Piercey 2022). The hanging wall and footwall sequences comprise mostly felsic volcanic rocks (FA signatures, Zr > 550 ppm). The sequence hosting the massive sulfide mineralization varies in thickness between 45 and 120 m (average ~ 100 m) and comprises interbedded felsic (FB signatures, Zr < 500 ppm) volcaniclastic rocks and minor argillites, coherent flows, sills, domes, and two mafic sills that extend through the deposit footprint.

The rocks in the ABM deposit footprint are hydrothermally altered (~ 1 km radius around the deposit), particularly the felsic lithofacies (Denisová and Piercey 2023). Proximal to mineralization, high-temperature pervasive chlorite assemblages (~ 315 °C) overprint lower-temperature pervasive sericite assemblages (~ 250 °C) or moderate sericite ± chlorite assemblages (~ 215 °C; Denisová and Piercey 2023). Mineralization overprints all types of pervasive alteration, although locally massive sulfides are contemporaneous with pervasive chlorite alteration. Pervasive alteration and mineralization occurred when the hydrothermal system at the ABM deposit was at its peak during a protracted break in volcanism that is recorded by a change in the felsic lithogeochemical signature and by the deposition of an argillite lens that is not affected by hydrothermal alteration.

The rocks of the Kudz Ze Kayah formation have been affected by greenschist facies metamorphism, recorded by mineral assemblages present in the mafic sills (chlorite–epidote–amphibole) and felsic volcanic rocks (white mica–chlorite). Primary bedding (S_0_) is recognized in argillite and mafic tuff of the Wind Lake formation; S_1_ that is subparallel to S_0_ is observed throughout the upper Kudz Ze Kayah formation in argillite and strongly altered units with abundant mica and chlorite (van Olden et al. 2020). Minor S_2_ folds and crenulation occur within argillites and rocks with a higher degree of hydrothermal alteration in both formations, but these are not indicative of any large-scale patterns on a deposit scale (van Olden et al. 2020). The deformation affected the pervasively altered zones the most, and mineralized zones show lesser degrees of deformation due to the abundance of pyrite (van Olden et al. 2020).

## Methods

Over 10 km of drill core from 50 drill holes were logged for this study. Graphic logging (scale 1:400) tracked lithology, primary textures, grain size, mineralogy, and alteration type and intensity based on mineral occurrence (quartz, white mica, chlorite, biotite, carbonates, and sulfides) and to document the sulfide mineralogy, textures, and relationships to host rock. Fifty-one samples representative of massive sulfide assemblages distributed across the ABM deposit were studied with a transmitted and reflected light petrographic microscope and a JEOL JSM 7100F scanning electron microscope (SEM) with backscattered electron (BSE) imaging operating at an accelerating voltage of 15 kV at the Hibernia Electron Beam Facility at Memorial University of Newfoundland (MUN). Selected polished thin sections of massive sulfide mineralization were imaged using SEM coupled with energy-dispersive X-ray spectroscopy (EDX) using a FEI MLA 650FEG instrument equipped with dual Bruker 5th generation XFlash SDD X-ray detectors at the Micro Analysis Facility at MUN-CREAIT, to show the semi-quantitative distribution of elements in areas with complex intergrowth textures. A dataset of all available assay data in the ABM deposit and surrounding areas was provided by BMC Minerals Ltd.; quality assurance and quality control (QA/QC) procedures for the company datasets are described in van Olden et al. (2020). Additional datasets provided by BMC Minerals Ltd., including core photos and drill logs, were used as secondary resources.

Digital models of mineralized lenses, alteration zones, and lithostratigraphic units displayed in the sections from the ABM deposit herein were created using the Leapfrog 2021.2 software. Isosurfaces representing the distribution of elements of interest were created using the assay database provided by BMC Minerals Ltd. and modeled using the Numeric Models tool in Leapfrog 3D. The linear radial basis function (RBF) interpolation was chosen to mitigate the irregular distribution of the datapoints, and it was run with a base range of 60, nugget of 0, and varying total sill and accuracy (Electronic Supplementary Material 1) for all the modeled isosurfaces. The trend for the numeric models was set to the local stratigraphy (dip 30° with dip azimuth of 20° and pitch of 115°); the ellipsoid ratios were set to 3:3:1.

### Electron probe microanalyzer

The compositions of pyrite, pyrrhotite, arsenopyrite, chalcopyrite, sphalerite, galena, and tennantite–tetrahedrite–freibergite in 15 polished thin sections were determined at Memorial University using the JEOL JXA-8230 SuperProbe electron probe microanalyzer (EPMA) equipped with five wavelength-dispersive spectrometers (WDS) and a tungsten filament electron gun. Natural and synthetic standards were used for calibration of the instrument, where the following standards and X-ray lines were used on five respective crystals (spectrometers), average detection limits for each element are given in parentheses: (1) LIF: sphalerite (Zn*K*α; 283 ppm), rhodonite (Mn*K*α; 150 ppm), pentlandite (Ni*K*α; 231 ppm); (2) PETL: stibnite (Sb*L*α; 50 ppm), silver (Ag*Lα*; 47 ppm), cadmium (Cd*L*α; 31 ppm), cinnabar (Hg*M*α; 65 ppm), bismuth (Bi*M*α; 115 ppm), galena (Pb*Mα*; 151 ppm), pyrite (S*Kα*; 32 ppm); (3) TAP: arsenopyrite (As*Lα*; 105 ppm), selenium (Se*L*α; 110 ppm); (4) LIFH: cuprite (Cu*Kα*; 46 ppm), cobalt (Co*Kα*; 28 ppm), pyrite (Fe*Kα*; 41 ppm). Counting times for calibration were between 10 and 30 s on peaks and 5 and 15 s on backgrounds. Analyses of unknown minerals were performed using the same crystals as the calibration. Pyrite, pyrrhotite, and arsenopyrite were analyzed for nine elements (Zn, Sb, Ag, Pb, S, As, Cu, Co, and Fe); sphalerite was analyzed for six elements (Zn, Mn, Cd, Hg, S, Fe); chalcopyrite was analyzed for eight elements (Zn, Ag, Hg, Bi, Pb, S, Cu, and Fe); galena was analyzed for 10 elements (Zn, Sb, Ag, Hg, Bi, Pb, S, Se, Cu, Fe); and tennantite–tetrahedrite–freibergite were analyzed for 12 elements (Zn, Ni, Sb, As, Hg, Pb, S, Se, As, Cu, Co, and Fe). The sulfides were analyzed using an accelerating voltage of 25 kV, a 2-nA beam current, focused to 1 µm, with elemental counting times between 5 and 30 s. Internal standards were measured periodically to demonstrate their reproducibility. Sulfide analyses with totals falling outside the 100 ± 2 wt. % range were rejected. For galena, pyrite, and sulfosalts, due to the irregular surface of some of the grains, analyses with totals falling outside 100 ± 3 wt. % range were rejected. Analyses in Electronic Supplementary Material (ESM) 2 were normalized to 100%. All analyses, calculated atoms per formula unit (apfu) values, and QA/QC data are available in ESM 2.

### Laser ablation inductively coupled plasma mass spectrometry

In situ LA-ICP-MS spot analyses (*n* = 116) were performed using a GeoLas 193 nm excimer laser (Coherent) coupled to a Thermo Finnigan ELEMENT XR ICP-MS instrument at Memorial University on six polished blocks where each sample represented one of the main mineral assemblages. The ICP-MS was tuned for high sensitivity and a ThO/Th ratio of < 0.3%. Concentrations of selected elements using analyte masses of ^34^S, ^55^Mn, ^57^Fe, ^59^Co, ^60^Ni, ^65^Cu, ^66^Zn, ^69^ Ga, ^72^Ge, ^75^As, ^77^Se, ^107^Ag, ^111^Cd, ^115^In, ^118^Sn. ^121^Sb, ^125^Te, ^197^Au, ^202^Hg, ^205^Tl, ^206^Pb, and ^209^Bi were determined for pyrite, pyrrhotite, sphalerite, galena, chalcopyrite, arsenopyrite, and tennantite. Ablation employed a spot diameter of 20 µm for galena and 30 µm for all other sulfides at a repetition rate of 5 Hz with an energy density of 3 J/cm^2^. For each spot, a gas blank was analyzed for 30 s, followed by 40 s of ablation. The standards NIST 610 (synthetic glass) and MASS-1 (pressed powder pellet) were measured every 20 analyses. NIST 610 was used for drift correction, and MASS-1 was used for calibration/matrix correction. Data reduction and the subtraction of gas blanks were performed using Iolite v. 3.72 (Paton et al. 2011); this program was used for data treatment, to inspect the time-resolved signals and to exclude time-resolved sections of the signal representing micro-inclusions. Detection limits and standard deviations for all analyzed elements, together with the collected data are available in ESM 3. Average values for a reference element in each mineral as determined using EPMA in each sample were used as internal ratio standards (Fe for pyrite, pyrrhotite, and arsenopyrite; Zn for sphalerite; Cu for chalcopyrite and tennantite; and Pb for galena). The mass ^115^In (natural abundance 95.72%) can show interferences from ^115^Sn (natural abundance 0.34%) but where the In concentrations are greater or similar to Sn concentrations, the interference effect on In by Sn is negligible.

## Observations and results

### Mineralization lenses extent, distribution, and morphology

**Fig. 3:**
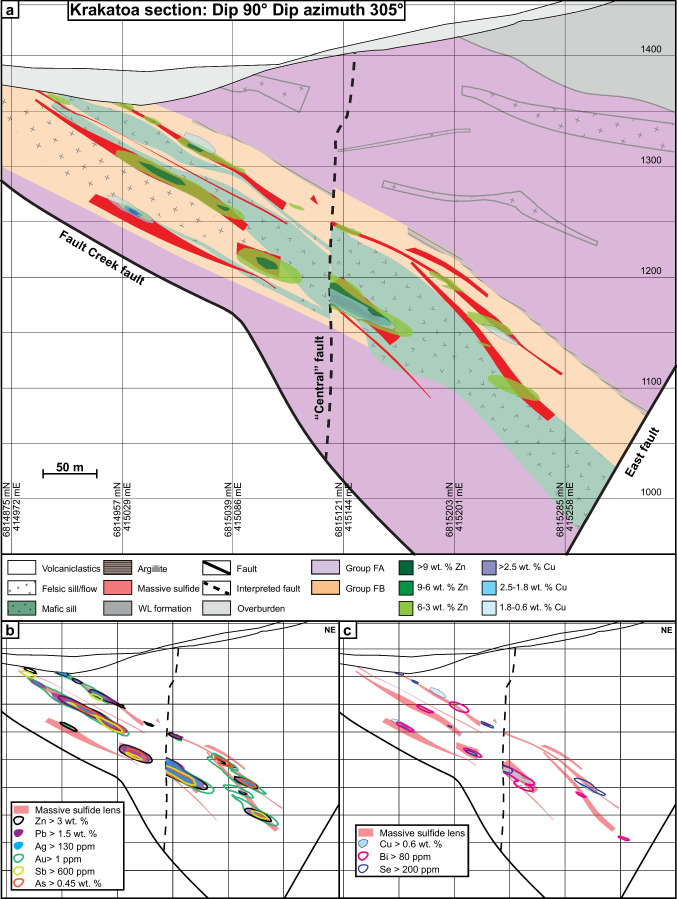
Cross section of the Krakatoa zone looking northwest. **a** Simplified lithostratigraphy of the Krakatoa zone with an overlay showing the distribution of elevated Zn and Cu. **b** Distribution of the Zn–Pb–Ag–Au–Sb–As element assemblage in the Krakatoa zone. **c** Distribution of the Cu–Bi–Se element assemblage in the Krakatoa zone

The ABM deposit contains two main mineralized zones — ABM and Krakatoa (Fig. 2a) — that were offset by ~ 200 m along the East fault post-mineralization. The mineralization in both zones consists of a series of stacked stratabound massive sulfide lenses that dip subparallel to the stratigraphy (20–30°; Figs. 2b and 3). The ABM zone is 700 m across and extends from surface down dip for 600 m. Mineralization in the ABM zone tapers off down dip to the NNE, along strike to the west, and is truncated by the East fault in the east; the thickness of the mineralization varies from 5 to 55 m. The western portion of the ABM zone has several thinner and less extensive massive sulfide lenses (at least seven lenses varying in thickness from < 1 to 10 m true thickness), some of which merge towards the east. The eastern portion of the ABM zone consists of a single thick (up to 20 m true thickness) massive sulfide lens. The Krakatoa mineralized zone is 170 m across and extends from surface down dip at least 600 m, and mineralized lenses are cut off by post-mineralization movement on bounding faults along strike in both directions (i.e., East fault and Fault Creek fault; Fig. 2a). The mineralized interval in the Krakatoa zone varies from 15 to 100 m in thickness. The Krakatoa zone is bisected by the post-mineralization “Central” fault, which offset the two blocks dextrally by at least a 100 m. The northern block contains thin massive sulfide lenses associated with a mafic sill, whereas the southern block contains most of the known mineralized lenses in the Krakatoa zone with true thickness varying up to 16 m (Fig. 3). In the Krakatoa zone, reactivated faults (e.g., “Central” fault) have cut through the mineralized zones and samples proximal to the fault show evidence of ductile deformation.

**Fig. 4:**
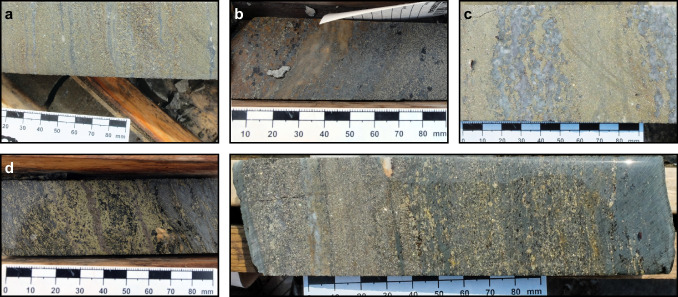
Replacement textures in massive sulfide mineralization at the ABM deposit. **a** Massive pyrite–sphalerite mineralization with remnant lapilli clasts with quartz crystals; clasts are white mica–chlorite altered; K15-274, 92 m downhole. **b** Pyrite replacing sericite-altered contact of felsic flow; K15-236, 97 m downhole. **c** Massive pyrite–sphalerite and minor chalcopyrite replacing a felsic flow along perlitic fractures; K15-200, 143 m downhole. **d** Banded pyrite–chalcopyrite–pyrrhotite mineralization with associated black chlorite replacing sericite–chlorite-altered felsic volcaniclastic rocks; K15-286, 127 m downhole. **e** Pyrite–sphalerite with mineralization with minor chalcopyrite replacing chlorite altered felsic volcaniclastic rocks; K15-235R, 140 m downhole. Scale in all photos is in millimeters

The ABM deposit is hosted by hydrothermally altered volcaniclastic and volcanic rocks. In the ABM zone, massive sulfide mineralization is associated primarily with felsic coherent and volcaniclastic rocks (Fig. 2b). In the Krakatoa zone, massive sulfide mineralization is localized on contacts between the mafic sills and felsic volcaniclastic rocks or, locally, within the mafic sills themselves (Fig. 3). Massive sulfide lenses in both zones generally have sharp contacts, although rarely they grade into altered rocks over a distance of 1–2 m. Features such as preserved lapilli and other clasts (Fig. 4a), remnant bedding (Fig. 4b), and massive sulfides replacing glassy groundmass within perlitic and brecciated textures at unit contacts (Fig. 4c) occur within the massive sulfide lenses and on their contacts suggesting that the mineralization formed, in part, by subseafloor replacement (van Olden et al. 2020; Denisová and Piercey 2022; Manor et al. 2022a).

### Mineral assemblages

In both the ABM and Krakatoa zones, massive sulfide mineralization consists of pyrite, locally abundant sphalerite and/or chalcopyrite, and lesser pyrrhotite, magnetite, galena, minor tetrahedrite group minerals, and rare sulfosalts and other minerals. The most common non-sulfide gangue minerals are barite, carbonate, quartz, chlorite, and white mica. Massive sulfide assemblages contain > 60 modal % of sulfides. The three main mineral assemblages (Table 1) are (1) pyrite–sphalerite with lesser galena, chalcopyrite, and tetrahedrite group minerals, with carbonate, barite, quartz, and white mica; (2) pyrite–chalcopyrite–magnetite–pyrrhotite with lesser sphalerite, minor tetrahedrite group minerals, and minor carbonate and chlorite; and (3) chalcopyrite-pyrrhotite-pyrite stringers associated with pervasive chlorite alteration, minor carbonate, and quartz.

**Table 1:** Mineralization assemblages in the ABM deposit

Assemblage	Major mineral (> 20 modal %)	Minor minerals (< 20 modal %)	Trace minerals (< 1 modal %)	Gangue minerals	Dominant element assemblage
Assemblage 1	Pyrite	Galena (< 3 wt. % Se)	magnetite	Barite	Zn–Pb–Ag–Au–Hg–As–Sb–Ba
40–60% of massive sulfide lenses	Sphalerite (< 5 wt. % Fe)	Chalcopyrite	Tennantite	Carbonate	
		Pyrrhotite	Tetrahedrite	Quartz	
		Arsenopyrite	Freibergite	White mica	
			Cassiterite		
Assemblage 2	Pyrite	Magnetite	Tetrahedrite	Quartz	Cu–Bi–Se
35–40% of massive sulfide lenses	Chalcopyrite	Pyrrhotite	Freibergite	Carbonate	
		Sphalerite (> 7 wt. % Fe)	Arsenopyrite		
		Galena (> 3 wt. % Se)			
Assemblage 3		Chalcopyrite	Galena (> 3 wt. % Se)	Chlorite	Cu–Bi–Se
10–15% of massive sulfide lenses		Pyrite		Carbonate	
		Pyrrhotite		Quartz	
		Magnetite			
		Sphalerite (> 7 wt. % Fe)			

#### Pyrite–sphalerite assemblage (assemblage 1)

**Fig. 5:**
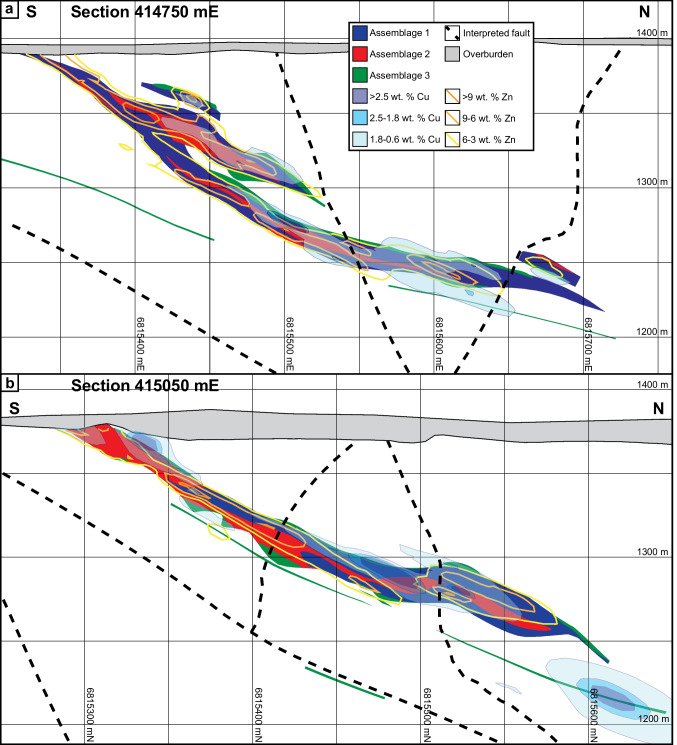
Cross sections through the eastern part of the ABM zone showing the distribution of mineral assemblages and of Cu and Zn values. (a) Section along the line 414750 mE looking west. (b) Section along the line 415050 mE looking west

**Fig. 6:**
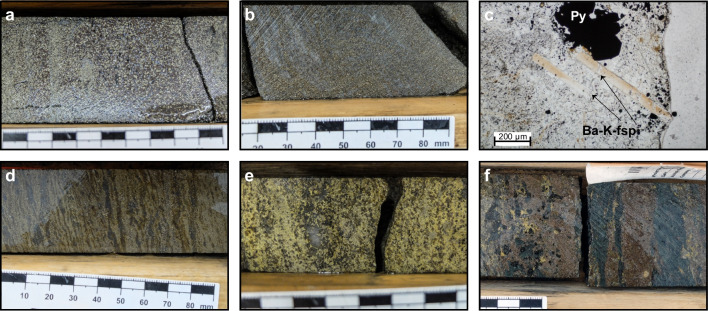
Mineral assemblages at the ABM deposit. **a** Buckshot pyrite texture in pyrite–sphalerite mineralization with abundant galena; K15-260, ~ 169 m downhole. **b** Pyrite-sphalerite assemblage with abundant associated barite; K15-232, 161 m downhole. **c** Elongated prismatic crystals of Ba-rich feldspar in a carbonate–barite matrix with disseminated fine-grained sulfides and clusters of euhedral pyrite grains; K15-236, 86.35 m downhole. **d** Banded pyrite–chalcopyrite–magnetite–pyrrhotite assemblage, magnetite appears as dark discontinuous lenses within pyrite–pyrrhotite bands, chalcopyrite minor; K15-274, 62 m downhole. **e** Massive pyrite–chalcopyrite–magnetite–pyrrhotite assemblage, minor associated black chlorite in matrix, rare quartz patch; K15-273, ~ 92 m downhole. **f** Pyrrhotite–chalcopyrite bands with black chlorite pseudomorphs replacing pervasively chlorite altered felsic volcaniclastic rocks; K17-422, 150 m downhole. Scale in all photos except for **c** is in millimeters. Ba-K-fsp = Ba-K-feldspar, Carb = carbonate, Gal = galena, Po = pyrrhotite

The pyrite–sphalerite assemblage is most common in the massive sulfide lenses and comprises ~ 45–50 vol. % of the total massive sulfide mineralization at the ABM deposit. The assemblage typically occurs on the lens margins (Fig. 5) and has sharp contacts with the surrounding altered rocks. Contacts with other assemblages are commonly gradational, although sharp contacts with pyrrhotite-rich intervals occur locally. Pyrite–sphalerite assemblages are commonly banded, with centimeter- to decimeter-scale bands that vary in composition (dominantly pyrite, sphalerite, barite, or carbonate bands) and/or grain size (Fig. 6a). Pyrite is the dominant sulfide in this assemblage, locally occurs in massive intervals, and is very fine- to relatively coarse-grained (up to the millimeter scale) and locally has buckshot textures, where granoblastic pyrite occurs within massive sphalerite (Fig. 6a). Sphalerite is commonly dark red to brown and fine-grained. Other sulfides (galena, chalcopyrite, arsenopyrite, tennantite–tetrahedrite, rare magnetite) occur in medium- to coarse-grained patches, locally associated with gangue minerals or remnant clasts, and/or in bands with sphalerite. Barite is the most common gangue mineral and occurs as diffuse layers within the mineralization (Fig. 6b). Locally, euhedral grains of Ba-rich feldspars occur (Fig. 6c); hyalophane (K-Ba-feldspar) is more common than celsian (Ba-feldspar), but celsian can be replaced by hyalophane along fractures, or rarely, both Ba-rich feldspars replace and/or overgrow K-feldspar. Rare cassiterite occurs as very fine-grained (< 10 µm) anhedral grains that are replaced along contacts by stannite. Remnant sericite and/or chlorite-altered lapilli-sized clasts (Fig. 4a) that are locally quartz-rich or replaced by carbonate occur within this assemblage. Where remnant clasts are abundant, they are aligned with the sulfide-defined banding (Fig. 4a, d, e).

#### Pyrite–chalcopyrite–magnetite–pyrrhotite assemblage (assemblage 2)

This assemblage comprises roughly 35–40% of the total massive sulfide mineralized zones at the ABM deposit and commonly occurs in the center of the individual massive sulfide lenses, surrounded by assemblages 1 and 3 (Fig. 5). Contacts between the assemblages are typically gradational over 10–50 cm, with a modal increase in chalcopyrite and/or magnetite towards assemblage 2. The assemblage is commonly banded, with centimeter- to decimeter-scale bands of pyrite, chalcopyrite, sphalerite, pyrrhotite, and locally magnetite (Fig. 6d). There are also massive intervals with abundant chalcopyrite and/or pyrrhotite (Fig. 6e). The bands vary in grain size, but where coarse, pyrite commonly displays a buckshot texture. Anhedral fine-grained chalcopyrite commonly occurs as patches and stringers, or in bands with pyrite and pyrrhotite. Magnetite is euhedral to subhedral, up to 0.5 cm in size, and occurs as patches or centimeter-scale bands of individual magnetite grains. Fine-grained to very fine-grained pyrrhotite occurs in bands and patches, commonly associated with chalcopyrite. Remnant clasts are typically quartz-rich and less common than in assemblage 1.

#### Chalcopyrite–pyrrhotite–pyrite stringer assemblage (assemblage 3)

Chalcopyrite–pyrrhotite–pyrite stringers occur within intervals of pervasive chlorite alteration and comprise ~ 10–15% of the total mineralization. The most common sulfides are chalcopyrite, pyrite, and pyrrhotite, with minor sphalerite or galena, and rare individual magnetite grains or patches. Carbonate and quartz are associated locally with the sulfides in bands and patches. This mineral assemblage occurs on contacts of the massive sulfide lenses (Fig. 5), or, less commonly, it transitions gradually into the pyrite–chalcopyrite–magnetite–pyrrhotite assemblage with decreasing chlorite content. The assemblage can also transition gradually to background pervasive chlorite alteration distal from the massive sulfide lenses. Assemblage intervals are commonly under 1.5 m thick, but locally, in the absence of other mineral assemblages, they extend up to 4 m in true thickness. The matrix comprises very fine-grained chlorite, while sulfides associated with lesser gangue minerals (carbonate, quartz) occur as bands or stringers on a centimeter to decimeter scale (Fig. 6f).

### Mineral textures

Minerals listed in the previous section (except for barite, Ba-rich silicates, Sn minerals, and less common sulfosalts and other rare minerals) occur across all mineral assemblages, even though they are too fine-grained and/or occur in too low abundances to be observed in drill core. In the following section, mineral textures will be described based on their assumed origin (and through literature comparison), including those that reflect (1) relict primary textures, (2) replacement features, (3) modified textures due to post-VMS metamorphism and deformation, or (4) mixed or of unknown origin.

#### Relict primary textures

**Fig. 7:**
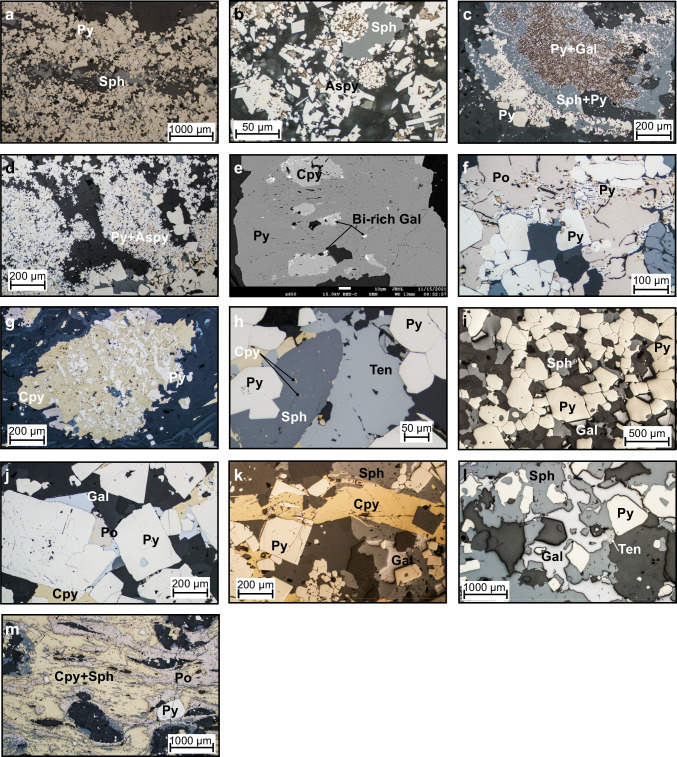
Mineral textures occurring at the ABM deposit. **a** Bands with varying grain size of pyrite, minor sphalerite, arsenopyrite, and galena present; K15-236, 86.4 m downhole. **b** Framboids comprising euhedral to subhedral arsenopyrite grains, infilled by galena; K15-321, 237.9 m downhole. **c** Atoll texture, pyrite at the core of the atolls is engulfed by galena, surrounded by sphalerite with only minor very fine-grained pyrite the rim comprises euhedral pyrite grains coarsening outwards; K15-236; 86.4 m downhole. **d** Spongiform pyrite and arsenopyrite, minor associated sphalerite, overgrown euhedral to subhedral pyrite; K15-231. 71 m downhole. **e** Minute Bi-rich galena exsolutions in chalcopyrite replacing pyrite; K15-204, 122.6 m downhole. **f** Fine-grained pyrite containing minute inclusions of Fe oxides and Fe carbonates replaces pyrrhotite, overprinted by euhedral pyrite; K15-231, 63.1 m downhole. **g** Skeletal pyrite replaced by chalcopyrite; K15-286, 127.1 m downhole. **h** Chalcopyrite disease in sphalerite; K15-229, 63.6 m downhole. **i** Foam texture, euhedral to subhedral fused pyrite grains, minor sphalerite, and galena in between grains; K15-229, 76.4 m downhole. **j** Euhedral pyrite grains with fine-grained inclusions in the cores; K15-339, 171 m downhole. **k** Fractured euhedral pyrite grain infilled by chalcopyrite; K15-339, 171 m downhole. **l** Subhedral pyrite grains engulfed by sphalerite, galena, and minor tennantite–tetrahedrite; K15-260, 171.8 m downhole. **m** Rounded euhedral grains of pyrite in massive chalcopyrite with sphalerite and pyrrhotite schlieren; K15-292, 239.7 m downhole. Aspy = arsenopyrite, Cpy = chalcopyrite, Cub = cubanite, Gal = galena, Po = pyrrhotite, Py = pyrite, Sph = sphalerite, Ten = tennantite

Numerous primary textures preserved in the ABM deposit have features that are similar to modern seafloor massive sulfide (SMS) deposits (Ames et al. 1993; Grant et al. 2018) and those found in well-preserved and relatively undeformed ancient VMS deposits (Eldridge et al. 1983; Martin et al. 2021). In assemblage 1, banding and finer-grained laminations interpreted to be primary occur as millimeter to centimeter layers defined by varying sulfide mineralogy and grain size (Fig. 7a). Within these layers, fine-grained pyrite and to a lesser extent arsenopyrite and sphalerite show relict primary textures, including rare round clusters of fine-grained to very fine-grained pyrite and arsenopyrite with framboidal features that are up to 50 µm across (Fig. 7b) and associated with galena and/or sphalerite. Similarly, fine-grained pyrite grains commonly constitute the cores of atoll textures (Fig. 7c). In these atolls, very fine-grained pyrite and/or arsenopyrite at the core is replaced/surrounded by galena and/or tennantite–tetrahedrite, which is then surrounded by sphalerite with only minor very fine-grained pyrite, and then a rim composed of coarse euhedral pyrite grains. These atolls locally fuse together or fuse with adjacent spongiform pyrite and/or arsenopyrite or are surrounded by gangue minerals in sulfide-poor bands or patches. Spongiform pyrite and arsenopyrite most commonly contain interstitial galena, sphalerite, and minor chalcopyrite and/or tennantite–tetrahedrite, and locally form bands or nodules within the massive mineralization. The spongiform pyrite–arsenopyrite bands commonly have margins where the spongiform sulfides are overgrown by coarser euhedral pyrite (Fig. 7d). Locally, very fine-grained elongated arsenopyrite grains occur as skeletal intergrowths in sphalerite.

In assemblages 2 and 3, relict primary textures are more subtle than in the pyrite–sphalerite assemblage. Locally, chalcopyrite overprints large subhedral pyrite grains (> 500 µm) and contains minute (< 2 µm) Bi-Se-bearing galena inclusions on the contacts with pyrite (Fig. 7e), similar to what has been observed in chalcopyrite-rich chimneys in SMS deposits and are interpreted to have formed due to rapid quenching of hydrothermal fluids (Berkenbosch et al. 2012).

#### Replacement textures

Replacement textures interpreted to be from zone refining and primary VMS hydrothermal processes are ubiquitous in assemblages 1 and 2. During continued zone refining, grain size coarsens (Eldridge et al. 1983). Similarly, in all assemblages, fine-grained anhedral pyrite is overgrown by coarser euhedral pyrite grains. Common throughout all the assemblages is the conversion of pyrrhotite into pyrite along cleavage planes, fractures, and grain boundaries (Fig. 7f). The fine-grained pyrite commonly contains minute inclusions of Fe oxides and Fe carbonates (Murowchick 1992). In assemblage 2, pyrite replaced by chalcopyrite commonly displays skeletal texture (Fig. 7g). Locally, minute chalcopyrite inclusions occur in anhedral sphalerite and in places they are aligned (Fig. 7h), which is indicative of chalcopyrite disease, a replacement feature common during the primary stages of VMS deposit formation (Barton and Bethke 1987).

#### Metamorphic textures

The ABM deposit has reached greenschist facies metamorphic grade and was also affected by deformation locally associated with the reactivation of synvolcanic faults (van Olden et al. 2020; Denisová and Piercey 2022). In all assemblages, bands and pyrite-rich zones commonly exhibit foam textures with 120° angles between the euhedral grains, where pyrite grains are annealed (Fig. 7i) interpreted to be from the impacts of increasing temperature and pressure (Craig and Vokes 1992). Other sulfides, originally surrounding the pyrite grains, are found as inclusions within the annealed mass, interpreted to have been trapped during metamorphic pyrite growth (Fig. 7j). In all assemblages, coarser euhedral pyrite grains (> 100 µm) locally display inclusion-free rims and inclusion-rich cores (Fig. 7j) that were likely originally spongiform and were overgrown and infilled during continued hydrothermal activity and/or metamorphism. Locally, pyrite displays a cataclastic texture with other sulfides infilling the cracks in the pyrite grains. Magnetite grains are commonly fractured, as well, but are not infilled by other sulfides as commonly as fractured pyrite; minor finer pyrite grains locally overgrow magnetite. Euhedral pyrite grains and to a lesser degree other sulfides (pyrrhotite, sphalerite) are commonly fractured, and the fractures are infilled by chalcopyrite (Fig. 7k). In the Krakatoa zone, in proximity to reactivated faults, slightly rounded euhedral grains of pyrite and carbonate clasts are interpreted to have rotated in massive chalcopyrite with sphalerite and pyrrhotite schlieren, or in massive sphalerite with galena schlieren (Fig. 7l, m).

#### Textures of unknown origin

**Fig. 8:**
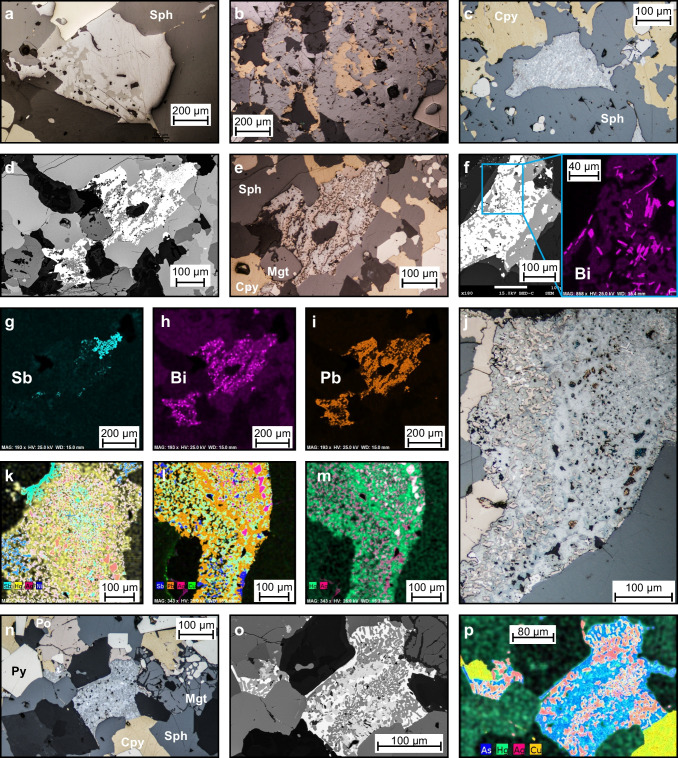
Symplectic intergrowths of unknown origin in the ABM deposit. **a** Galena with associated tennantite and tetrahedrite; K15-303, 212.8 m downhole. **b** Anhedral chalcopyrite in tennantite, minor associated galena; K15-231, 56. 1 m downhole. **c** Semi-parallel bands of meneghinite, bournonite, and tetrahedrite in symplectic intergrowth; K15-292, 239.7 m downhole. **d** Backscattered electron (BSE) image of symplectic intergrowth comprising pyrrhotite, Bi- and Se-enriched galena and gudmundite; K15-231, 63.1 m downhole. **e** Reflected light image of **d**. **f** BSE image of a symplectic intergrowth comprising Se- and Bi-enriched galena and pyrrhotite, with a close-up of an EDX elemental map showing the distribution of Bi as discrete patches within the intergrowth; K15-204, 112.6 m down hole. **g**–**i** EDX elemental maps showing the distribution of Sb, Bi, and Pb, respectively, in the symplectic intergrowth pictured in **d** and **e**. **j** Symplectic intergrowth showing mineralogical and elemental zonation comprising pyrrhotite, galena, freibergite, unknown Ag–Hg–Sb mineral, meneghinite, and gudmundite; K15-292, 239.7 m downhole. **k** Composite image of EDX elemental maps of Sb, Hg, Ag, and Ni for the top portion of **j**. **l** Composite image of EDX elemental maps of Sb, Pb, Ag, and Cu for the bottom portion of **j**. **m** Composite image of EDX elemental maps of Hg and Ag for the bottom portion of **j**. **n** Symplectic intergrowth comprising freibergite, meneghinite, boulangerite, and galena; K15-339, 171 m downhole. **o** BSE image of **n**. **p** Composite image of EDX element maps of As, Hg, Ag, and Cu for **n**. Cpy = chalcopyrite, Gal = galena, Po = pyrrhotite, Py = pyrite, Sph = sphalerite

Assemblages 1 and 2 are characterized by 100–300-µm (up to 1 mm) clusters of intergrown minerals that have uncommon mineral associations and are of uncertain origin. The clusters in assemblage 1 have two mineral associations: (1) galena–tennantite–tetrahedrite and (2) chalcopyrite–tennantite–tetrahedrite. The first association occurs as anhedral patches of galena with irregular patches of tennantite–tetrahedrite and other lesser sulfosalts (e.g., boulangerite). These intergrowths have a symplectic appearance, and the patches appear to be later than or infilling between the surrounding pyrite and sphalerite grains (Fig. 8a). The second association displays anhedral patches of chalcopyrite in tennantite–tetrahedrite (Fig. 8b). These occur where chalcopyrite and tennantite–tetrahedrite coexist, associated with galena, and fractured euhedral pyrite. Previous authors (Bortnikov et al. 1993; Cook 1996) described similar textures and attributed them to decomposition due to changing As/Sb activities in the hydrothermal fluid, although the occurrence of these textures locally associated with fractured pyrite grains at the ABM deposit suggests the possibility of metamorphic origin (Miller and Craig 1983; Brueckner et al. 2016).

Assemblage 2 clusters contain intergrown galena (Se- and/or Bi-rich), pyrrhotite, Bi minerals (native Bi, bismuthinite), minor tetrahedrite–freibergite, Pb-rich sulfosalts (bournonite, boulangerite, meneghinite), Sb-rich sulfides (gudmundite, ullmannite), and rare Ag–Hg–Sb minerals. The non-tetrahedrite group sulfosalts and Sb-rich sulfides are fine- to very fine-grained and occur as anhedral grains within galena and/or pyrrhotite. Locally, they are intergrown with the base metal and Fe-bearing sulfides but have a less distinct “myrmekite-like’ appearance (Fig. 8d–i). In clusters larger than 100 µm, parallel bands of sulfosalts and Sb-rich sulfides, likely crystallographically oriented, occur within galena (Fig. 8c), and some clusters show a mineralogical zonation (Fig. 8j–p). The more complex of these intergrowths occur in the Krakatoa zone (Fig. 8c, g–p), although they show lower contents of Se and Bi than the clusters in the ABM zone (Fig. 8d–i).

#### Paragenesis

**Fig. 9:**
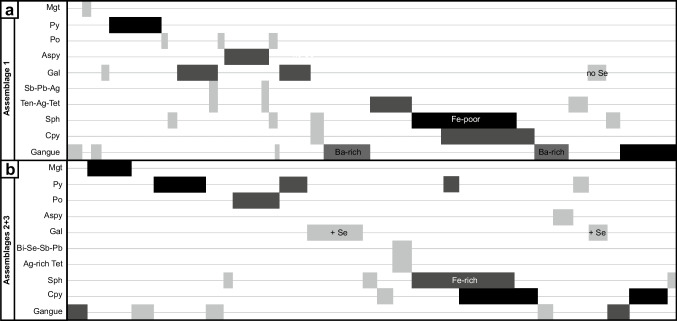
Apparent mineral paragenesis at the ABM deposit determined from preserved primary and replacement textures; effects of metamorphism are not shown. **a** Paragenesis for assemblage 1. **b** Combined paragenesis of assemblages 2 and 3. Black color of bars indicates modal abundances > 30%, dark gray indicates modal abundances between 30 and 10%, and light gray indicates modal abundances < 10%

Despite overprinting relationships, an “apparent” mineral paragenesis can be determined from preserved primary and replacement textures and their inter-relationships (Fig. 9). Assemblages 2 and 3 display similar relationships between the most abundant minerals and based on the observations from drill core, assemblage 2 overprints assemblage 1. The “apparent” paragenesis presented here is consistent across the ABM deposit.

Pyrite formation (fine-grained, commonly with atoll and spongiform textures) in assemblage 1 (Fig. 9a) was followed by galena and arsenopyrite precipitation, formation of barite and Ba-rich feldspar, tetrahedrite group minerals, and co-precipitation of abundant sphalerite with lesser chalcopyrite. The youngest minerals to form are calcite and Fe-rich carbonate. In assemblages 2 and 3 (Fig. 9b), the earliest observed mineral accompanying the silicate gangue minerals is magnetite. Magnetite grains are commonly sub- to euhedral with fractures filled by gangue minerals and overgrown by fine-grained pyrite. The early formed sulfides are dominated by pyrite and pyrrhotite, and were followed by the precipitation of galena, and abundant chalcopyrite with lesser co-precipitated sphalerite.

### Metal distribution and zonation in massive sulfide zones

Economically significant metals at the ABM deposit are Zn, Pb, Cu, Ag, and Au. Other metals and metalloids occurring within the mineralized zones are Fe, As, Sb, Se, Bi, Hg, Co, Ni, Mo, Tl, Cd, Sn, In, and Mn. The distribution and concentrations of these elements reflect which sulfides occur within the massive sulfide lenses. Although the above-described assemblages control the lens-scale enrichment of these metals, all assemblages may carry economic concentrations of Cu, Zn, and Pb (van Olden et al. 2020).

**Fig. 10:**
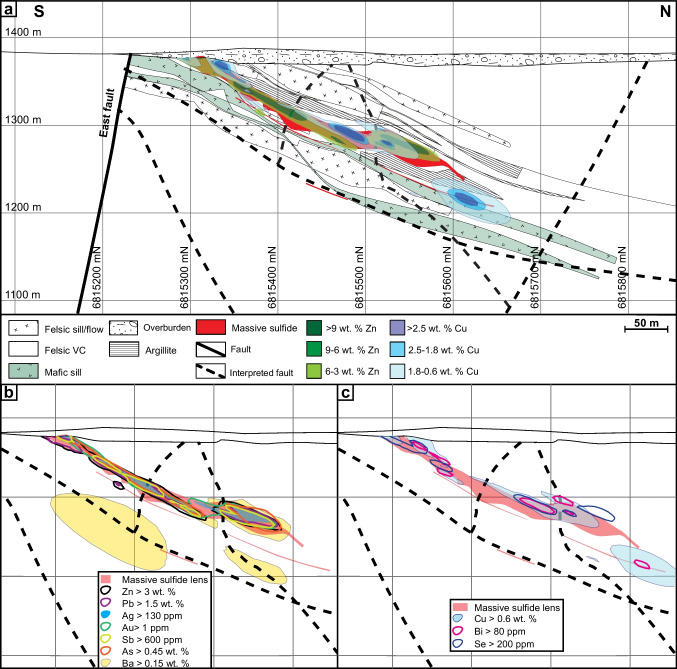
Cross section through the eastern part of the ABM zone along the line 415,050 mE looking west. **a** Simplified lithostratigraphy of the ABM zone with an overlay showing the distribution of elevated Zn and Cu. **b** Distribution of the Zn–Pb–Ag–Au–Sb–As element assemblage. **c** Distribution of the Cu–Bi–Se element assemblage

Massive sulfide mineralization in the ABM zone has Cu-rich zones (> 0.9 wt. % Cu) at the center of the mineralized lenses, which commonly extend to the upper contacts of the lenses (Figs. 2b, 3, and 10). Even where Cu-rich zones overlap with elevated Zn (> 6 wt. % Zn), the Zn-rich zones occur at the base of the mineralized lenses and extend further along the lenses (Figs. 2b and 10). In the Krakatoa zone, the Cu-rich zones are more limited vertically than in the ABM zone and do not reach the upper contacts of the lenses (Fig. 3). Across both zones, Pb is strongly associated with Zn but there is no distinguishable zonation developed between Pb and Zn on a deposit scale (Figs. 3 and 10). Zones with elevated Ba (> 1 wt. % Ba) locally overlap with and extend beyond the limits of Zn-Pb-rich zones (Fig. 10), with anomalous Ba values (> 0.15 wt. % Ba) extending beyond the massive sulfide mineralization into the altered host rocks (Denisová and Piercey 2022).

In assemblage 1, sphalerite is the primary Zn-bearing mineral. Cadmium and Hg commonly substitute in sphalerite and positively correlate with Zn in assay data (ESM 4). Zinc and Pb also have a broad positive correlation (ESM 4). Galena is the primary Pb-bearing mineral in assemblage 1, and only Ag shows a significant positive correlation with Pb (ESM 4). These correlations are reflected in the spatial distribution of the elements within the massive sulfide lenses (Figs. 3 and 10). Arsenic, Sb, and Ba show a spatial correlation with elevated Zn, Pb, and Ag (Figs. 3 and 10), but not a distinct correlation in the assay dataset. The observed distribution of As, Sb, and Ba within assemblage 1 correlate with increased arsenopyrite, tennantite–tetrahedrite, and barite in this assemblage compared to others.

In assemblage 2, chalcopyrite is the primary Cu mineral. Copper and Bi values do not correlate well in the assay dataset (ESM4); however, Se and Bi overlap Cu spatially in the ABM deposit (Figs. 3 and 10). Magnetite occurs in minor amounts within the assemblage (< 10 modal %), and magnetic monoclinic pyrrhotite (Kissin and Scott 1982) is locally more abundant than pyrite; these magnetite-enriched zones are common in the cores of massive sulfide lenses.

Assemblage 3 shows overall higher contents of Cu, Bi, and Se, compared to the other two assemblages, and contain lower Ba, Pb, Zn, Ag, Au, Hg, and As.

#### Element associations

Principal component analysis (PCA) using a correlation matrix performed on log-normalized bulk assay data shows two major element associations: (1) a Zn–Pb–Ag–Au–Hg–As–Sb–Ba association, which has positive loadings of component 1 and negative loadings on component 2, and (2) a Cu–Bi–Se association that has positive loadings on component 2 (ESM 5). These associations correspond to the overlapping spatial distribution of metals within the mineral assemblages in the massive sulfide lenses across the ABM and Krakatoa zones (Figs. 3 and 10). Numeric models representing elevated Ag and Au overlap each other, and with zones representing elevated As, Sb, Zn, and Pb values (Figs. 3 and 10).

### Electron microprobe analysis results

The complete EPMA results are available in ESM 2. The composition of the analyzed sulfides is generally stoichiometric, but there are systematic variations of mineral compositions depending on the mineral assemblage, paragenesis, or spatial distribution (ABM zone vs Krakatoa zone).

#### Base metal sulfide minerals

**Fig. 11:**
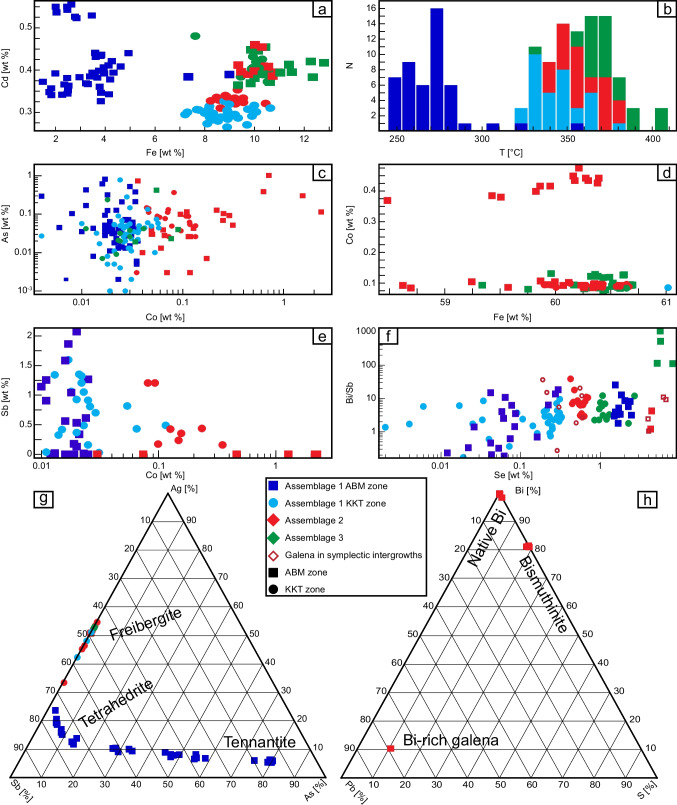
Results of EPMA analyses. Sample analyses with below detection values are excluded from the plots. **a** Fe vs Cd (both in wt. %) in sphalerite. **b** Histogram showing the distribution of calculated temperatures using equation from Keith et al. (2014). **c** Co vs As (both in wt. %) in pyrite. **d** Fe vs Co (both in wt. %) in pyrrhotite. **e** Co vs Sb (both in wt. %) in arsenopyrite. **f** Se vs Bi/Sb (Se in wt. %) in galena. **g** Ternary diagram showing Ag, Sb and As distribution in tetrahedrite group minerals. **h** Ternary diagram showing Bi, Pb, and S distribution in Bi-rich minerals

The Zn and S contents in sphalerite vary between 53.3 and 65.4 wt. % and between 32.6 and 37.1 wt. %, respectively. Sphalerite can be divided into two groups based on Fe content: low (< 5 wt. % Fe), and high (> 7 wt. % Fe), which are found in assemblage 1 in the ABM zone, and assemblage 2 throughout both deposit zones, respectively (Fig. 11a). Cadmium content is between 0.25 and 0.58 wt. %, but there is no correlation with other analyzed elements (Fig. 11a). Sphalerite from the ABM zone has a higher average Cd (0.41 ± 0.06 wt. % Cd, *n* = 76) than that from the Krakatoa zone (0.32 ± 0.05 wt. % Cd, *n* = 60).

Copper, Fe, and S contents in chalcopyrite vary between 30.8 and 34.8 wt. % Cu, 29.8 and 32.5 wt. % Fe, and 34.5 and 36.4 wt. % S. Silver is enriched (between 0.06 and 0.15 wt. %) in samples from assemblage 3 and assemblage 2 from the Krakatoa zone.

The Pb and S contents of galena vary between 61.7 and 86.9 wt. % and between 8.4 and 13.8 wt. %, respectively. Selenium content is up to 8.9 wt. % and varies systematically with the type of mineralization (Fig. 11f), where it is highest (> 3 wt. %) in assemblage 3 and assemblage 2 from the ABM zone. The same samples show elevated Ag (0.2–1.65 wt. %) and Bi (0.75–4.5 wt. %). In rare cases, galena in these assemblages contains up to 10 wt. % Bi (Fig. 11h). In the Krakatoa zone, the highest Se content is 2.75 wt. % Se, and samples from assemblage 1 have Se < 0.35 wt. % (Fig. 11f).

#### Iron sulfide minerals

The Fe and S contents in pyrite vary between 43.9 and 47.9 wt. % and between 52.4 and 54.3 wt. %, respectively. Assemblage 2 in the ABM zone has elevated Co values (> 0.1 wt. % Co; Fig. 11c), whereas elevated As (> 0.15 wt. % As) occurs in assemblage 1 in the ABM zone.

The Fe and S contents in pyrrhotite are between 58.7 and 60.3 wt. % and between 39.5 and 40.7 wt. %, respectively, consistent with monoclinic pyrrhotite (Kissin and Scott 1982). Pyrrhotite from sample Q721151 has significantly higher Co values (between 0.35 and 0.50 wt. % Co) similar to the pyrite in the sample. All pyrrhotite has Co > 0.05 wt. % (Fig. 11d).

Arsenic, Fe, and S contents in arsenopyrite vary between 40.3 and 44.3 wt. % As, 34.3 and 37.0 wt. % Fe, and 19.1 and 22.7 wt. % S, respectively. The highest values of Co (max 2.27 wt. %) occur in samples from assemblage 2 (Fig. 11e), whereas Sb (0.13–2.12 wt. %) is elevated in samples from assemblage 1.

#### Sulfosalts and Sb-rich sulfides

The most common sulfosalts occurring within the mineralization are tetrahedrite group minerals (tetrahedrite, tennantite, freibergite). In assemblage 1 in the ABM zone, tetrahedrite group minerals have up to 8.5 wt. % Ag (Fig. 11g) and significant Fe (ranging between 2.6 and 7.8 wt. %) and Zn (between 2.0 and 4.8 wt. %) that correlate inversely. Freibergite contains minimal As (less than 0.16 wt. %) but contains significant Pb (0.5–20.1 wt. %), Fe (4.5–9.8 wt. %), and lesser Zn (0.4–1.4 wt. %).

Other identified sulfosalts and Sb-rich sulfides are rare, commonly very fine-grained, and include bournonite, boulangerite, meneghinite, gudmundite, and ullmannite (ESM 2).

#### Bismuth minerals

In assemblage 2, rare (< < 1 modal %) minerals rich in Bi occur (Fig. 11h; ESM 2). Very fine-grained native bismuth grains can occur (> 90 wt. % Bi) and have minor Sb (between 1 and 3 wt. %) and trace Fe (< 1 wt. %) values. Bismuthinite (Bi_2_S_3_) also occurs; its Bi and S contents vary between 81 and 84 wt. % and between 7.8 and 19.1 wt. %, respectively, with minor Fe (between 1.3 and 3.3 wt. %).

### LA-ICP-MS results

The complete LA-ICP-MS results are available in ESM 3. Results from LA-ICP-MS are generally in accordance with the EPMA analyses, but document a more diverse suite of elements, particularly those at low concentrations. Gold contents range between 0.007 and 17.1 ppm; the highest values (> 1 ppm Au) occur in arsenopyrite (max 17.1 ppm), galena (max 6.7 ppm), chalcopyrite (max 2.5 ppm), and pyrite (max 2.5 ppm). The highest values of Hg occur in tennantite–tetrahedrite (> 50 ppm) and sphalerite (8–25 ppm). Highest values of Tl occur in galena (~ 30–350 ppm, locally up to 1224 ppm).

#### Base metal sulfide minerals

Sphalerite grains from samples of assemblage 2 show elevated contents of Se, Bi, and Co.

Chalcopyrite grains from assemblages 2 and 3 have elevated Se and Ag contents compared to chalcopyrite from assemblage 1. Samples in the Krakatoa zone from all assemblages are enriched in Ag compared to those from the ABM zone.

**Fig. 12:**
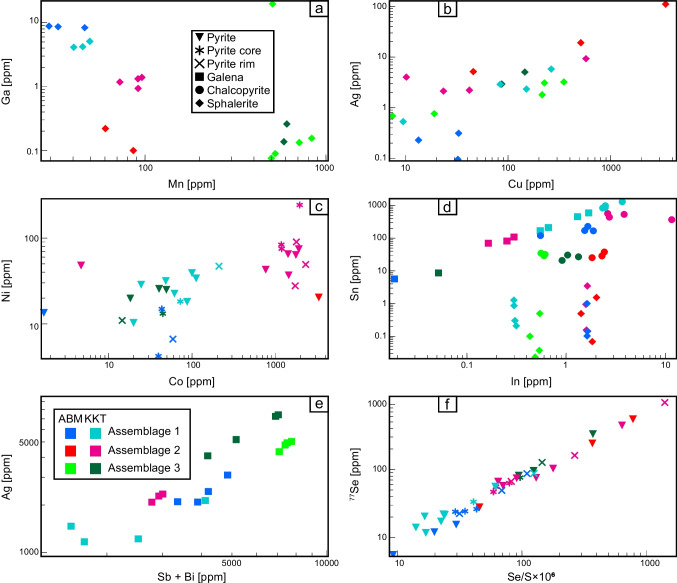
Results of LA-ICP-MS analyses. Sample analyses with below detection values are excluded from the plots. **a** Mn vs Ga in sphalerite. **b** Cu vs Ag in sphalerite. **c** Co vs Ni in pyrite. **d** In vs Sn in chalcopyrite, galena, and sphalerite. **e** Sum of Sb and Bi vs Ag in galena. **f** Se/S × 10^6^ vs Se in pyrite

Galena grains from assemblages 2 and 3 are enriched in Bi, Ag, and Se (Fig. 12e), whereas grains in assemblage 1 show elevated As, Fe, Tl, and Cd. Analyzed galena grains from the Krakatoa zone show elevated In and Sn regardless of mineral assemblage (Fig. 12d).

#### Pyrite

Pyrite grains from assemblages 2 and 3 have elevated contents of Se, Co, Ni, and As (Fig. 12c), whereas in assemblage 1 they have higher average Tl and Hg. Zoned pyrite grains commonly occur in all assemblages where cores of the grains display very fine-grained inclusions of other sulfide minerals and higher trace metal content compared to the inclusion free rims in all assemblages (Fig. 12c).

## Discussion

Some VMS deposits in orogenic belts present challenges in deciphering the relative roles of primary hydrothermal processes and effects of post-VMS metamorphism and deformation. In the following sections, the impact of metamorphic overprinting and how it has affected the primary geochemistry and mineralogy of the ABM deposit will be evaluated. This will then be contrasted with mineralogical, textural, and geochemical features that are primary and reflect the conditions of deposition during the formation of the replacement-style VMS mineralization. We will then compare the mineralization at the ABM zone and the Krakatoa zone to determine whether they belong to the same mineralizing system, including discussing the potential sources of metals and their enrichment in the ABM replacement-style VMS deposit.

### Effects of metamorphism and deformation

The ABM deposit has numerous features indicative of primary stratigraphy, lithofacies, and hydrothermal alteration that have been documented in the ABM deposit (e.g., Denisova and Piercey, 2022, 2023). Despite preservation of primary features, the deposit area has evidence for greenschist facies metamorphism, particularly distal from the mineralization footprint where the rocks have middle greenschist facies assemblages (e.g., chlorite, actinolite, epidote; Murphy et al. 2006). At these conditions, recrystallization of minerals is common (Lafrance et al. 2020) but with minimal impacts on the original chemistry of silicate minerals (Riverin and Hodgson 1980; Urabe et al. 1983; Hannington et al. 2003). In contrast, the mineralogy and mineral chemistry of sulfide minerals can be affected (Barton and Bethke 1987; Lockington et al. 2014; Kampmann et al. 2018); however, the scale of the effects varies with intensity of the metamorphism and deformation (Marshall et al. 1998), and the rheological properties of the contained sulfide minerals (Marshall and Gilligan 1987).

Arsenopyrite geothermometry can provide insight into the metamorphic conditions affecting the sulfides and is based on As content in arsenopyrite that is in equilibrium with other Fe sulfides and with < 1 wt. % Sb + Co + Ni (Kretschmar and Scott 1976; Sharp et al. 1985). Arsenopyrite commonly occurs fine-grained in spongiform patches together with pyrite (Fig. 7b, d), suggesting it formed coeval with pyrite which have both been affected by metamorphism. Similarly, where re-crystallized with pyrite and displaying foam textures (Fig. 7i), arsenopyrite with < 1 wt. % of Sb + Co + Ni has As content varying between 29 and 31.6 at. % (ESM 2), which corresponds to a temperature range of 300–420 °C (Kretschmar and Scott 1976), consistent with greenschist facies metamorphism. Textural features found in the sulfides are also consistent with greenschist facies metamorphism and deformation effects on the sulfides.

#### Effects of metamorphism and deformation on mineral textures at different scales

In the ABM deposit, macro-scale banding in the sulfide mineralization has features characteristic of tectonic banding (Lafrance et al. 2020), including monomineralic sulfide and polymineralic modal sulfide bands, and elongation of silicate fragments. The mineralization at the ABM deposit is interpreted to be replacement-style, so where sulfide minerals are interpreted to have replaced the volcaniclastic rocks, the S_0_ fabric of the volcaniclastic rocks should have been preserved within the sulfides. Previous studies (van Olden et al. 2020) documented that the main S_1_ fabric is subparallel to primary bedding S_0_ in host rocks to the ABM deposit. In the drill core, the orientation of the S_1_ bedding does not differ notably between the massive sulfide lenses and variously altered host rocks, which indicates that the tectonometamorphic processes have not fully erased the original fabric and that some of the existing macro-scale structures (bedding/banding, mineral assemblage distribution) in the massive sulfide mineralization are preserved primary features.

On the micro-scale, however, textures reflective of metamorphism and deformation are more pronounced and have features consistent with greenschist facies metamorphism and arsenopyrite geothermometry outlined above (Murphy et al. 2006; Denisová and Piercey 2023). For example, pyrite commonly has fractures infilled by chalcopyrite, galena, or sphalerite (Fig. 7k). Durchbewegung structures (Marshall and Gilligan 1989), where coarse pyrite grains occur in a matrix of fabric-defining chalcopyrite and sphalerite (Fig. 7l, m), occur locally. Similarly, symplectic-like clusters of galena and associated sulfosalts and Sb-rich sulfides within the pyrite–chalcopyrite–magnetite–pyrrhotite assemblage (Fig. 8c–p) are analogous to textures found in quenched sulfide melts (Tomkins et al. 2007). In particular, Tomkins et al. (2007) suggested that at these conditions, if elevated Bi, Hg, Sb, and/or As are present and multiple minerals (galena, chalcopyrite, arsenopyrite, sulfosalts) coexist in the mineral assemblages, sulfide anatexis can occur. The presence of elevated Bi in assemblage 2 galena and its spatial association with inclusions of bismuthinite and/or native Bi (Fig. 8f, h), as well as symplectic intergrowths of tetrahedrite, freibergite and Pb- and Pb–Sb-rich sulfosalts, and more rarely, sulfosalts with elevated metals and metalloids like Hg, Ni, Tl, and Se are consistent with partial melting of sulfides.

While sulfide partial melting occurred, the scale of partial sulfide melting observed at the ABM deposit is much smaller (clusters < 1 mm in size) and the intergrowths make up a negligible portion of the mineralization (< < 0.1 vol. %), compared to other deposits where the scale of partial melting is interpreted to be much larger (e.g., Broken Hill, Lengenbach; Hofmann 1994; Sparks and Mavrogenes 2005). This is likely due to the lower metamorphic grade (greenschist facies) and lower metamorphic temperatures affecting the ABM deposit (vs ~ 750–800 °C at Broken Hill; Sparks and Mavrogenes 2005). Moreover, symplectic intergrowths that are interpreted to be the products of sulfide partial melting are found only in zones of Bi enrichment (ABM zone) or in Cu-enriched zones where maximum deformation/strain has been observed (Krakatoa zone). Therefore, in the ABM zone, Bi-enrichment appears to be a key factor for initiating sulfide melting, but the effects of sulfide anatexis are negligible on the deposit scale and only operated on a micrometer to centimeter scale and did not affect the mineral and element assemblages at the ABM deposit on the macro to deposit scales.

#### Effects of metamorphism and deformation on sulfide mineral chemistry

Metamorphism and structural overprinting have clearly impacted the textures of mineralization in the ABM deposit; however, these features had minimal effects on the distribution of major elements in sulfide minerals, while trace element distributions have been variably affected. For example, pyrite grains that are > 100 µm in diameter show inclusion-rich cores with higher trace element contents, have primary fine-grained textures, and are interpreted to be primary VMS-related features, compared to inclusion-poor rims that are trace element poor and are interpreted to be due to metamorphic recrystallization (ESM 3). Sphalerite locally exhibits chalcopyrite disease, a feature common in primary VMS mineralization and is evidence for local preservation of primary VMS textures (e.g., Barton and Bethke 1987); however, most sphalerite is inclusion free with Cu < 600 ppm (Fig. 12b), implying elimination of micro-inclusions due to metamorphic recrystallization (Craig and Vokes 1992; Lockington et al. 2014; Cugerone et al. 2021). Further, Hg enrichment in sphalerite is highest of all mineral phases (except tennantite; ESM 3), which is typical for sphalerite that has undergone metamorphic recrystallization (Lockington et al. 2014).

Iron content in sphalerite has been used as a geothermometer and a geobarometer (Scott and Barnes 1971) but is only valid if the VMS mineralization has not been affected by metamorphism above lower greenschist conditions (Keith et al. 2014) or if it has not been metamorphosed above 310 °C, the closure temperature of the sphalerite trace element geothermometer (Frenzel et al. 2016). Using the equation from Keith et al. (2014), the calculated sphalerite temperatures at the ABM deposit show a bimodal distribution consisting of a lower temperature group (235–290 °C) representing sphalerite with low Fe content in the pyrite–sphalerite assemblage from the ABM zone of the deposit, and a higher-temperature group (320–410 °C) representing Fe-rich sphalerite from all other mineral assemblages in both zones (Fig. 11b). Using the Frenzel et al. (2016) trace element thermometer for sphalerite yields temperatures between 300 and 380 °C for samples from assemblages 2 and 3 (*n* = 8), and temperatures between 223 and 281 °C for grains (*n* = 7) from assemblage 1 (ESM 3). The temperatures in the first group are typical for similar sphalerite-rich assemblages in modern SMS systems; however, some calculated temperatures in the second group are too high, as sphalerite typically forms at ~ 290 ± 50 °C in SMS and VMS systems (Pisutha-Arnond and Ohmoto 1983; Halbach et al. 1993). In modern SMS systems, hydrothermal fluid can reach temperatures > 350 °C (Hannington et al. 2005) and precipitate assemblages with abundant chalcopyrite and pyrrhotite, and minor sphalerite, which are similar to assemblages 2 and 3 in the ABM deposit. However, the high calculated temperatures for the pyrite–sphalerite assemblage 1 in the Krakatoa zone suggest that at least in some samples, the Fe and Zn contents in sphalerite (and the resultant geothermometer temperatures) were modified by zone refining or by post-VMS metamorphism.

Trace element distributions were also affected by metamorphic recrystallization. During metamorphic recrystallization, if multiple sulfides (galena, sphalerite, and chalcopyrite) co-crystallize or recrystallize simultaneously, they acquire trace element signatures that are distinct from those typical for precipitation from hydrothermal fluids (George et al. 2016; Kampmann et al. 2018). For example, in co-crystallized assemblages, Sn is preferentially enriched in chalcopyrite followed by galena and sphalerite (George et al. 2016, 2018), and Ga and In prefer chalcopyrite over sphalerite (George et al. 2016, 2018). The mineral scale distribution of Sn, Ga, and In conforms to these trends (Fig. 12d) and suggests that chalcopyrite, galena, and sphalerite have been, in part, recrystallized during metamorphism. This distribution of trace elements between phases is consistent across mineral assemblages, albeit with absolute trace element contents in the minerals varying between the assemblages (e.g., elevated Bi, Se, and Co in assemblages 2 and 3, or enrichment in Sb, As, Cd, In, and Ga in assemblage 1). Regardless of absolute concentrations, the mineral-scale distributions described above support mineral-scale trace element redistribution during metamorphism of the massive sulfide mineralization at the ABM deposit.

### Conditions during the precipitation of the replacement-style VMS mineralization at the ABM deposit

While greenschist facies metamorphism affected some textures and trace element distributions in the ABM deposit, there are windows in the deposit that have undergone less strain and metamorphism, and where primary textures are preserved (e.g., spongiform and atoll textures, framboids). In these zones, sulfide mineral chemistry and lens-scale geochemical trends can be used to determine the primary conditions of massive sulfide formation. In VMS deposits, mineral assemblages and mineral chemistry of sulfide mineralization reflect the original temperature, redox, and pH conditions of the fluids that the mineralization precipitated from (Large 1977; Solomon and Walshe 1979; Lydon 1988; Ohmoto 1996; Franklin et al. 2005). The following section provides insights into the potential primary depositional conditions during the formation of the ABM deposit, and the relative differences in precipitation conditions between the ABM and Krakatoa zones in the ABM deposit.

**Fig. 13:**
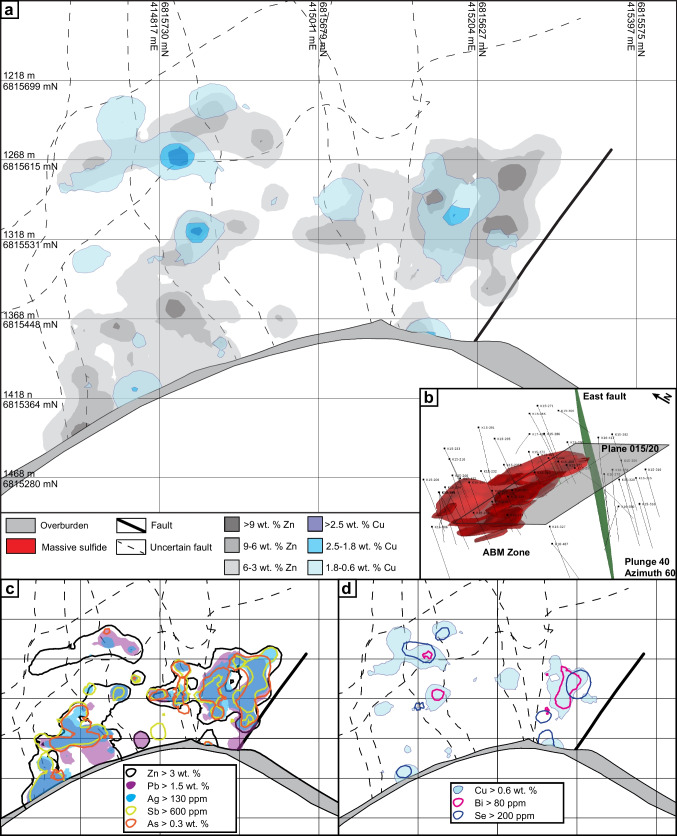
View along section parallel to main trend of massive sulfide mineralization (section plane 015/20) in the ABM zone. **a** Plan view of the section showing the cross section of the massive sulfide lenses and distribution of Cu and Zn. **b** Oblique view of the ABM zone showing where the viewing plane cuts the massive sulfide mineralization. **c** Distribution of the Zn–Pb–Ag–Sb–As element assemblage. **d** Distribution of the Cu–Bi–Se element assemblage

Zone refining, the dissolution and re-precipitation of ore and gangue minerals, generates a temperature-dependent metal zonation in long-lived thermally evolving VMS deposits (Eldridge et al. 1983; Lydon 1988). In replacement-style deposits, metal zonation follows the same sequence as in exhalative- and mound-style deposits with interiors dominated by Cu and exterior portions of the lenses being enriched in Zn, Pb, and Ba (Knuckey et al. 1983; Lydon 1988), albeit with different geometries that reflect the porosity and permeability of the host rocks that controlled the subsurface hydrothermal fluid flow. In particular, the metal zonation is more pronounced laterally than vertically in replacement-style mineralization (Bradshaw et al. 2008; Piercey et al. 2014; Nozaki et al. 2021). The metal distribution within the massive sulfide mineralization in the ABM zone has Cu-rich zones at the center of the mineralized lenses, overlapping with Zn-rich zones at the margins of the lenses (Figs. 2b, 10, and 13). Zones that are enriched in Cu and associated metals (Bi–Se–Co) generally have a similar trend to some of the interpreted synvolcanic faults (Fig. 13d; Denisová and Piercey 2022), suggesting the faults likely acted as conduits for ascending high temperature hydrothermal fluids. As Cu is commonly the metal to precipitate from hydrothermal fluids at the highest temperatures (Pisutha-Arnond and Ohmoto 1983), this trend in Cu enrichment is also interpreted to delineate the zones where the fluids infiltrated laterally along synvolcanic faults into the porous and permeable host units (Fig. 13).

Compared to Cu, the distribution of Zn–Pb–Ba extends further from the synvolcanic structures and into the surrounding host rocks (Figs. 10b and 13c). In general, Pb is associated with Zn, and locally zones with elevated Ba (> 1 wt. % Ba) overlap with Zn–Pb-rich zones and extend into altered host rocks with Ba values > 0.15 wt. % (Fig. 10b; Denisová and Piercey 2022). Despite the effects of metamorphism and deformation described above, the deposit-scale metal zonation (inner Cu → Zn–Pb → Ba outwards) at ABM appears not significantly affected by them and has a zonation typical for VMS deposits (Knuckey et al. 1983; Lydon 1988; Large 1992). This deposit-scale metal zonation and the character and distribution of mineral assemblages also likely resulted from changing temperature, pH, and redox conditions during deposition and are evaluated more fully below.

#### Evidence for low-temperature (< 270 °C) fluids and seawater mixing

Generally, in SMS and VMS deposits, assemblages enriched in Zn–Pb–Ba with abundant pyrite, sphalerite, and barite are interpreted to have formed at temperatures of 250 ± 50 °C from the mixing of acidic and reduced hydrothermal fluids with seawater (Pisutha-Arnond and Ohmoto 1983). Tennantite–tetrahedrite and barite commonly occur in the low-temperature and more distal parts of VMS and SMS deposits (Grant et al. 2015), whereas pyrrhotite is typical for high-temperature assemblages proximal to the center of the hydrothermal system (Knuckey et al. 1983; Large 1992). Mineral textures in low-temperature assemblages are commonly fine-grained, due to rapid nucleation resulting from fluid mixing, and include framboids, colloform, spongiform, and atoll textures (Butler and Rickard 2000).

At the ABM deposit, assemblage 1 (pyrite-sphalerite) is the most voluminous, occurs at the margins of the massive sulfide lenses, roughly outlines the extent of the Zn–Pb–As–Sb–Ag–Au–Hg–Ba enrichment zones, and represents zones where the majority of arsenopyrite, tennantite–tetrahedrite–freibergite, and barite occur, and pyrrhotite is minor. In this assemblage, low-temperature (200–270 °C) textures include atoll and spongiform textures and rare framboids with pyrite and/or arsenopyrite (Fig. 7b–d). The abundance of arsenopyrite in assemblage 1 suggests potential precipitation from reduced hydrothermal fluids (Heinrich and Eadington 1986). In contrast, tennantite and tetrahedrite, likely paragenetically younger than arsenopyrite, also occur in assemblage 1 but require more oxidized conditions to precipitate (Grant et al. 2015). Additionally, tetrahedrite forms later than tennantite and locally replaces it. Sulfosalts overprinting arsenopyrite is more prevalent in the Krakatoa zone, where freibergite, which requires even more oxidizing conditions to precipitate than tetrahedrite (Grant et al. 2015), commonly occurs. The decomposition textures locally associated with the tetrahedrite group minerals (Fig. 8a, b) suggest that during the evolution of assemblage 1 there was increasing oxidation of the fluids, which rendered the various tetrahedrite-group minerals unstable.

Sulfide mineral chemistry also provides insights into the depositional conditions of the mineralization. Despite metamorphism, Co, Ni, Se, and Te that originally substituted into the mineral lattice of sulfide/sulfosalt minerals during VMS formation are generally not affected by greenschist facies metamorphism and reflect primary VMS formation conditions (Huston et al. 1995). Cobalt in pyrite is proportional to pyrite depositional temperature (Huston et al. 1995) and that the Co concentrations are lower in assemblage 1 pyrite (Fig. 12c) suggests this assemblage formed at lower temperatures than the other assemblages; this is also consistent with the framboidal, atoll, and spongiform textures noted in assemblage 1. Similarly, Bi–Sb–Se systematics of galena also favor a lower-temperature origin. For galena to carry more than 0.1 wt. % of Ag in solid solution, Bi and/or Sb also have to be present and balance out the Bi + Sb:Ag ~ 2:1; Sb is more abundant at lower temperatures than Bi (Amcoff 1984; Foord and Shawe 1989). Further, Se in galena is also governed by temperature, with Se substituting for S in the mineral lattice (Amcoff 1984; Huston et al. 1996). In both the ABM and Krakatoa zones, galena in assemblage 1 has lower Se compared to the other two assemblages, and while Ag values in galena can be up to 0.38 wt. % (Fig. 12e), Ag substitution is offset by Sb substitution instead of Bi (low Bi/Sb; Fig. 11f). These trends in galena composition suggests a lower temperature of galena formation in assemblage 1 compared to other assemblages. These features are also paralleled by the Fe content in sphalerite, which is lower in assemblage 1 compared to other assemblages and suggests precipitation from lower temperature (*T* < 300 °C) and less reduced fluids, at least for the ABM zone (1.5–6 wt. % Fe).

Collectively, these data illustrate that in assemblage 1, the mineralogy, textures, and behavior of greenschist facies metamorphism-resistant elements suggest that assemblage 1 formed at relatively lower temperatures than assemblages 2 and 3, and that the redox conditions of the environment varied during the precipitation of the assemblage 1 (Fig. 9a), where minerals that commonly precipitate under more reducing conditions (e.g., pyrrhotite, arsenopyrite) preceded those that require relatively more oxidizing conditions to precipitate (tetrahedrite group minerals, Fe-poor sphalerite).

#### Evidence for high-temperature (270–350 °C) reducing fluids

In modern and ancient deposits, assemblages containing abundant chalcopyrite and pyrrhotite are interpreted to have formed at temperatures between 300 and 360 °C (Pisutha-Arnond and Ohmoto 1983; Hannington et al. 2005). Locally, primary textures indicative of high-temperature precipitation and zone refining in other VMS deposits (Eldridge et al. 1983; Craig and Vokes 1992), such as chalcopyrite disease in sphalerite, skeletal pyrite, or pyrrhotite replacing pyrite (Fig. 7f–h), are also preserved at the ABM deposit, which implies that chalcopyrite-rich mineralization was deposited during high-temperature (*T* > 300 °C) primary VMS hydrothermal activity.

Pyrrhotite is locally abundant (up to 33 modal %) and occurs early in the paragenesis of assemblages 2 and 3 (Fig. 9). Pyrrhotite is commonly replaced along fractures and grain boundaries by fine-grained “ribbed” pyrite (Fig. 7f), and this is interpreted to record the influx of younger, more oxidized fluids that dissolved the pyrrhotite and replaced it with pyrite (Murowchick 1992; Grant et al. 2015). The average pyrrhotite composition (59.80 ± 0.29 wt. % Fe, *n* = 77; ESM 2) matches that of monoclinic pyrrhotite (Kissin and Scott 1982), and while monoclinic pyrrhotite can form by direct precipitation from hydrothermal fluids, it commonly occurs at temperatures < 258 °C (Lianxing and Vokes 1996), which is at the lower end of the temperature range suggested by the chlorite thermometer (Denisová and Piercey 2023) and other sulfides common in assemblages 2 and 3. Locally, pyrrhotite displays annealed textures (Fig. 8n), which are typical in metamorphosed deposits (Craig and Vokes 1992), and this suggests that annealing and transformation from hexagonal to monoclinic pyrrhotite were thorough during metamorphism. Thus, pyrrhotite in assemblages 2 and 3 was likely originally hexagonal and formed at temperatures higher than > 272 °C, and was subsequently recrystallized during metamorphism, or possibly during zone refining as the deposit evolved.

Other sulfide mineral chemical attributes in assemblages 2 and 3 are indicative of high-temperature deposition. For example, pyrite from assemblage 2 has elevated Se, Co, and Ni compared to the assemblage 1 (Figs. 11c and 12c), indicative of formation at higher temperatures (Huston et al. 1995; Genna and Gaboury 2015; Martin et al. 2021). Elevated Se content in pyrite (Fig. 11f), and in the minor galena occurring in this assemblage compared to assemblage 1 (Fig. 11f), is also an indicator of high (> 300 °C) temperatures of precipitation under reducing conditions (Huston et al. 1995; Layton-Matthews et al. 2008). Further, galena in assemblages 2 and 3 has elevated Bi/Sb values (Fig. 12f) and Ag values up to 1.25 wt. %, which are also indicative of higher temperatures and lower redox conditions compared to assemblage 1 (Amcoff 1984; Grant et al. 2015). This is also mirrored by Fe content in sphalerite in assemblage 2 (9–11 wt. %), which is associated with pyrite and similar to the values from assemblage 3, both of which implies that the hydrothermal fluids forming these assemblages were likely more reducing than those forming assemblage 1 (e.g., Scott 1983; Keith et al. 2014).

The stringer assemblage 3 is not extensive and commonly occurs on the margins of the massive sulfide lenses where it transitions into the unmineralized host rocks with pervasive chlorite alteration assemblages (Fig. 5). Formation temperatures calculated for chlorite in both mineralized and barren pervasive chlorite assemblages are ~ 275–345 °C (Denisová and Piercey 2023). Pyrrhotite and chalcopyrite commonly occur in this assemblage, and minor sphalerite has relatively high Fe content (~ 9–13 wt. %) and elevated Mn content (Fig. 12a) compared to other assemblages at the ABM deposit, which is typical for sphalerite that precipitated from reduced hydrothermal fluids (Scott 1983; Keith et al. 2014; Frenzel et al. 2016). The recorded temperatures for the pervasive chlorite hydrothermal alteration assemblage also correspond to temperatures recorded for similar, chlorite–chalcopyrite-rich assemblages in modern SMS and ancient VMS deposits (Pisutha-Arnond and Ohmoto 1983; Large 1992; Hannington et al. 2005). The occurrence of assemblage 3 predominantly on the margins of the massive sulfide lenses suggests it formed together with the pervasive chlorite alteration assemblage from reduced fluids with some of the highest temperatures reached in the mineralizing system.

#### Relationship between the ABM and Krakatoa zones

The relationship between massive sulfide mineralization in the ABM and the Krakatoa zones of the ABM deposit is not fully understood. Despite post-mineralization offset along the East fault, the mineralization occurs in a similar stratigraphic position in both zones, which implies contemporaneous development of the massive sulfide lenses (Denisová and Piercey 2022). The predecessor of the East fault was a major structure that controlled basin subsidence and likely acted as a pathway for upwelling VMS hydrothermal fluids (Denisová and Piercey 2022). Reconstruction of the offset along the East fault suggests that the two zones were not connected directly, because the number and characteristics of the mineralized lenses do not match between the two zones (Denisová and Piercey 2022). In the Krakatoa zone, the mafic sills take up more volume within the mineralization-hosting sequence and have greater control over the distribution of the massive sulfide mineralization compared to the ABM zone (Figs. 2 and 3). The mafic sills are interpreted to have had significantly lower porosity and permeability, in contrast to the surrounding volcaniclastic rocks and are interpreted to have acted as barriers to hydrothermal fluid flow and limited the influx of seawater into the more porous and permeable lithofacies in their footwall.

The mineralogical and element assemblages of the ABM and Krakatoa zones are generally very similar; however, their distribution and extent vary. Ohmoto (1996) and Hannington et al. (1998) suggested that if zone refining in VMS and SMS deposits continues to its full course, lower-temperature assemblages will be on the margins of the massive sulfide lenses and the mineralized bodies can eventually become fully pyritized. In the ABM zone, the distribution of assemblage 2 reaches all the way to the hanging wall contact at the center of the massive sulfide lenses (Figs. 5 and 10c). In the Krakatoa zone, assemblage 1 is more extensive and the distribution of assemblage 2 is vertically more limited than in the ABM zone (Fig. 3), suggesting that the hydrothermal system was possibly less active than in the ABM zone, either due to barriers to fluid flow, or the hydrothermal fluid flow was active for a shorter period in the Krakatoa zone. The recorded differences in mineralogy and mineral chemistry between the two zones are relatively minor, such as the more common occurrence of freibergite in the Krakatoa zone, which suggests a greater influence of oxidized fluids in this zone, likely due to more mixing with seawater given the possibly less vigorous hydrothermal fluid flow.

Given their very similar mineralogy, mineral chemistry, and element assemblages, the mineralization at the ABM zone and the Krakatoa zone likely shared the same underlying source of hydrothermal fluids but the depositional conditions varied due to differences in host rock facies and their distribution, which controlled the influx of seawater and the upwelling of the high temperature hydrothermal fluid.

### Metal sources and genetic model

The formation of the massive sulfide mineralization at the ABM deposit was an evolving process, where earlier assemblages were overprinted and replaced by later ones. The earliest deposition of sulfide minerals in the massive sulfide lenses was likely euhedral magnetite grains common in assemblage 2 and rare in assemblage 1 (Fig. 9). The grains likely precipitated before the hydrothermal fluid became more reduced and/or rich in H_2_S, possibly during the formation of early hydrothermal alteration assemblages. This was followed by continuous and extensive infiltration of hydrothermal fluids that were moderate temperature (200–270 °C), rich in H_2_S, acidic and reduced, into the subsurface along porous and permeable units, where the hydrothermal fluids interacted with infiltrated seawater (Denisová and Piercey 2022, 2023) and precipitated assemblage 1. As the system heated up (> 270 °C), higher-temperature hydrothermal fluids ascended along the synvolcanic faults, permeated assemblage 1, and, through zone refining processes, dissolved existing Zn- and Pb-rich phases and precipitated chalcopyrite in their stead, leading to the deposition of assemblage 2 in the centers of the massive sulfide lenses, and with the Zn- and Pb-rich fluids derived from dissolution being reprecipitated on the margins of the existing sulfide lenses. With time and continued infiltration of high-temperature hydrothermal fluids, assemblage 2 grew outward from the synvolcanic faults, which resulted in the dissolution and reprecipitation of assemblage 1 outward into the host rocks. Zones of assemblage 3 within and on the contacts of the massive sulfide lenses are likely a result of limited high-temperature (> 350 °C) pulses of hydrothermal fluids that formed together with the zones of pervasive chlorite alteration (Fig. 5). The wide distribution of the hydrothermal alteration assemblages (Denisová and Piercey 2023) and the considerable tonnage and grade contained by the deposit (van Olden et al. 2020) suggest that the hydrothermal system that formed the deposit was potentially robust and long-lived (e.g., up to ~ 400 k.y.; Denisová and Piercey 2022; Manor et al. 2022a).

The mineral assemblages at the ABM deposit are enriched in element assemblages that have been attributed by some to magmatic-hydrothermal fluids in both VMS and SMS deposits (Sillitoe et al. 1996; Hannington et al. 1999a; Sillitoe and Hedenquist 2003; de Ronde et al. 2005). In assemblage 1, the As–Sb–Hg–Ag–Au element association and the locally abundant tetrahedrite group minerals and barite are consistent with an arc- or intermediate sulfidation-type assemblage (Sillitoe and Hedenquist 2003). The enrichment of Cu–Se–Bi–Co in assemblage 2 has characteristics similar to other deposits where a magmatic–hydrothermal contribution to the mineralization has been suggested (e.g., the bornite zone in the Kidd Creek VMS deposit; Hannington et al. 1999b). However, other characteristics typical for magmatic–hydrothermal VMS deposits are lacking. The only potential intrusion that could have contributed magmatic–hydrothermal fluids mapped in the area (so far) was emplaced after the formation of the ABM deposit (Manor et al. 2022b). Further, in assemblage 1, minerals that typically form under reducing conditions (arsenopyrite, Fe-rich sphalerite in the Krakatoa zone) are common, and the mineral assemblages suggest precipitation from low *f* O_2_ hydrothermal fluids, which is atypical for magmatic–hydrothermal fluids (Sillitoe and Hedenquist 2003). Additionally, in magmatic–hydrothermal environments, the strongly acidic nature of fluids commonly forms high-Al alteration assemblages containing alunite or pyrophyllite (Hannington et al. 2003; Sillitoe and Hedenquist 2003); such alteration assemblages are not preserved at the ABM deposit. Despite this, the presence of abundant illite in the pervasive sericite hydrothermal alteration assemblage (Denisová and Piercey 2023) could have potentially formed because of dilution of the extremely acidic magmatic–hydrothermal fluids by mixing with abundant seawater.

At present, the arguments above for a direct magmatic–hydrothermal contribution to the hydrothermal fluids that formed the ABM deposit are permissive, but circumstantial. In particular, it is possible that the presence of magmatic–hydrothermal-like element and mineral assemblages may be due to the leaching of rocks with magmatic–hydrothermal metal assemblages like those discussed above (Lydon 1988; Kase et al. 1994; James et al. 2003; Franklin et al. 2005). Previous studies demonstrated that Se in sulfides can be used to track the origin of hydrothermal fluids (Huston et al. 1995; Layton-Matthews et al. 2008, 2013). At the ABM deposit, only galena and sulfides from assemblages 1 and 2 in the ABM zone show Se/S × 10^6^ values > 1000 (ESM 3). Most of the sulfides have signatures that are different from those associated with magmatic–hydrothermal origins (Huston et al. 1995; Layton-Matthews et al. 2008), which, coupled with published Se isotope data from ABM, are consistent with leaching of basement rocks of potentially magmatic or volcanic origin (Layton-Matthews et al. 2013). Layton-Matthews et al. (2013) also suggested that the source of Pb in the massive sulfide mineralization at the ABM deposit was the leaching of basement of Laurentian affinity, and Mortensen et al. (2006) showed from Pb isotopes that basement leaching was important in most VMS systems along the western Laurentian margin. Moreover, the western margin of Laurentia contains numerous shale basins, including parts of the Finlayson Lake district; thus, it would be reasonable to assume that these could have been potential sources of metals for VMS hydrothermal systems. Black shales can be enriched in elements like Co, Bi, Se, Cu, Zn, As, Ag, Tl, and Sb, depending on their depositional environment (Vine and Tourtel 1970; Hatch and Leventhal 1992; Brumsack 2006; Paikaray 2012) and trace elements commonly occur in sulfides and/or are associated with organic molecules and would potentially have been available for leaching (Vine and Tourtel 1970; Paikaray 2012). Leaching of sedimentary rocks, and black shales in particular, or of volcanic rocks, could also potentially account for the enrichment of metals with magmatic-hydrothermal affinity (e.g., As–Sb–Hg–Ag–Au and/or Cu–Se–Bi–Co) in the ABM deposit; however, this requires further study to decipher fully.

## Conclusions

Textural, mineralogical, and assay data show that the effects of greenschist facies metamorphism at the ABM deposit are limited to recrystallization, small-scale remobilization (< 1 m), and trace element redistribution. Deposit-scale metal zonation Cu → Zn-Pb → Ba corresponds to the distribution of mineral assemblages and reflects lowering temperatures and more oxidizing conditions as hot, reduced, metal-rich hydrothermal fluids infiltrated porous substrate, mixed with cold seawater, and precipitated ore minerals, which were subsequently modified by zone refining as the deposit matured. The widespread pyrite–sphalerite mineral assemblage zones (assemblage 1) coincide with the Zn–Pb–Ag–Au–Hg–As–Sb–Ba element association and formed at temperatures ~ 200–270 °C under fluctuating redox conditions. Assemblage 1 includes varying amounts of arsenopyrite, tetrahedrite group minerals, and barite. Pyrite–chalcopyrite–magnetite–pyrrhotite assemblage zones (assemblage 2) occur in the centers of massive sulfide lenses and overlap with zones of Cu–Bi–Se–Co enrichment. Assemblage 2 formed at temperatures ~ 300–350 °C, which is illustrated by commonly occurring chalcopyrite, pyrrhotite, Fe-rich sphalerite, and rare arsenopyrite. The similarities in mineral textures, mineralogy, and trace metal enrichment signatures between the ABM and Krakatoa zones suggest they were part of the same hydrothermal system, yet the differences in the distribution of the mineral assemblages indicate that the hydrothermal system was active for a longer period of time in the ABM zone than in the Krakatoa zone. Element associations characteristic of the observed mineral assemblages are reminiscent of deposits with direct magmatic–hydrothermal contributions to the hydrothermal fluids; however, the hydrothermal alteration assemblages and the sulfide mineral chemistry suggest that leaching of volcanic and/or magmatic rocks was the major metal source for the ABM deposit, even though a direct magmatic–hydrothermal contribution cannot be completely excluded.

### Supplementary Information

Below is the link to the electronic supplementary material.

Supplementary file1 (XLSX 11 KB)

Supplementary file2 (XLSX 201 KB)

Supplementary file3 (XLSX 49 KB)

Supplementary file4 (PDF 2571 KB)

Supplementary file5 (XLSX 294 KB)
