# Supplementary material for: Mineralogy and mineral chemistry of the ABM replacement-style volcanogenic massive sulfide deposit, Finlayson Lake district, Yukon, Canada
Source: Miner Depos. 2023 Oct 6;59(3):473–503. doi: 10.1007/s00126-023-01217-4 (PMC10861405; doi:10.1007/s00126-023-01217-4)

Electronic supplementary material 4
Figure: Plots showing correlations between metals at the ABM deposit
Article: Mineralogy and mineral chemistry of the ABM replacement-style volcanogenic massive sulfide deposit, Finlayson Lake district, Yukon, Canada
Authors: Nikola Denisova, Stephen Piercey, Markus Walle
Corresponding author: ndenisova@mun.ca

ESM Fig 4. Plots showing correlations between metals at the ABM deposit. “Streak” artifacts at low concentrations result from different detection limits and decimal point rounding as values approach detection limits. (a) Zn vs. Cd. (b) Zn vs. Hg. (c) Zn vs. Pb. (d) Pb vs. Ag. (e) Cu vs. Bi. (f) Cu vs. Co

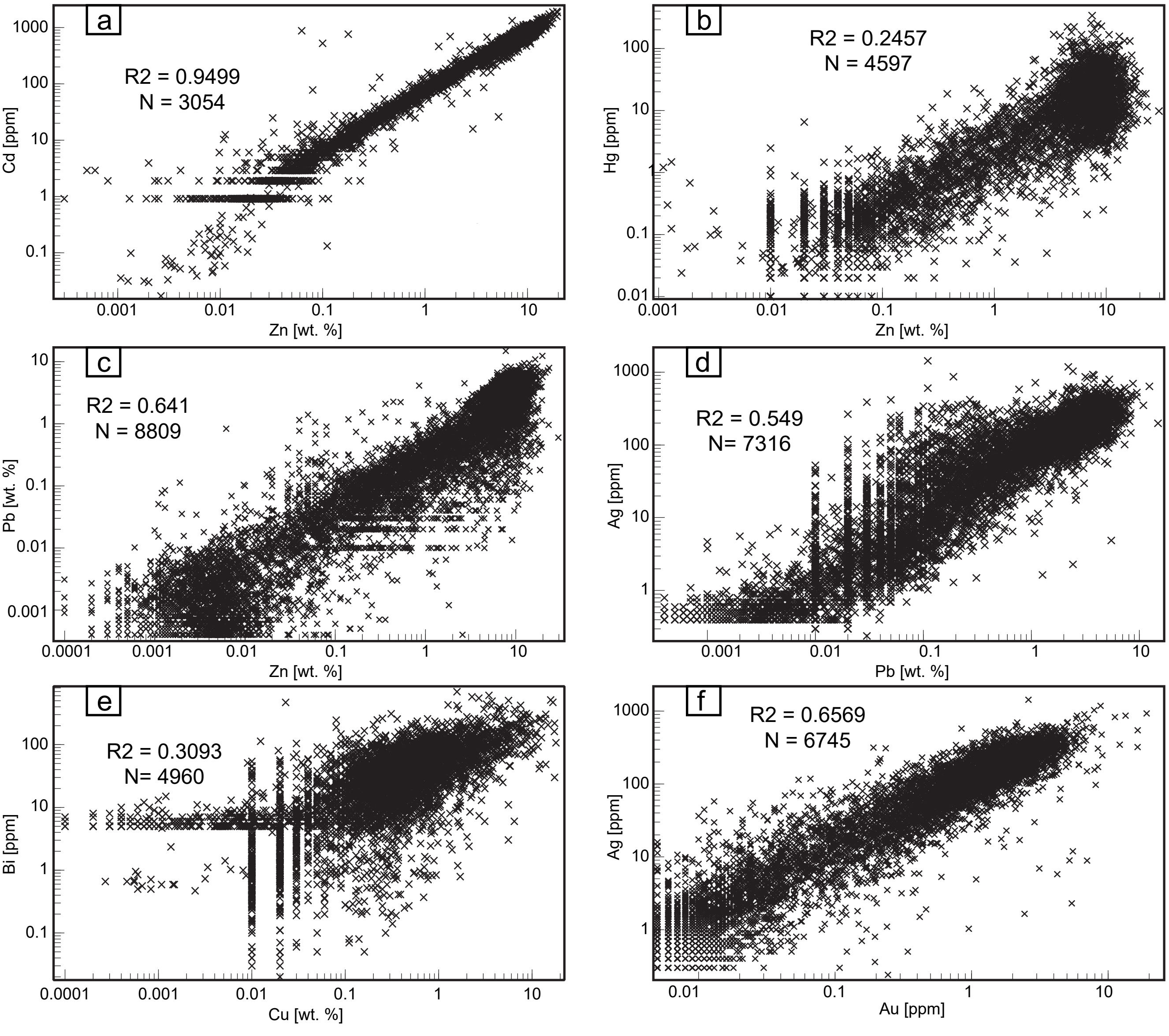

Supplement: Supplementary file 4 — Supplementary file4 (PDF 2571 KB) [file 126_2023_1217_MOESM4_ESM.pdf]
